# Mechanical properties of rooted soil under freeze–thaw cycles and extended binary medium constitutive model

**DOI:** 10.1038/s41598-023-40845-8

**Published:** 2023-08-21

**Authors:** Wei Luo, Bo Xiang, Enlong Liu, Haisong Zhao, Kai Wu, Yunyong He

**Affiliations:** 1 State Key Laboratory of Hydraulics and Mountain River Engineering, College of Water Resources and Hydropower, Sichuan University, Chengdu, 610065 China; 2 Sichuan Provincial Transport Department Highway Planning, Survey, Design and Research Institute, Chengdu, 610041 Sichuan China

**Keywords:** Civil engineering, Mechanical properties

## Abstract

In seasonally frozen soil, soil sometimes is affected by freeze–thaw cycles and root systems. In order to study its mechanical characteristics, a series of consolidation drained triaxial tests under different confining pressures (25, 50, 100, 200 kPa), different freeze–thaw cycles (N = 0, 1, 5, 15) and different root-containing conditions (r = 0, 1, 3) were carried out. The test results show that the specimens exhibit strain softening behavior and volumetric dilatancy phenomena and shear failure under lower confining pressure, and strain hardening and volumetric contraction, bulging failure under higher confining pressure. With the increase of freeze–thaw cycles, the bearing capacity of the sample decreases and the volume strain increases. With the increase of volume ration of roots in the sample, the bearing capacity increases and the volume strain decreases. Based on the binary medium model, the soil is abstracted into bonded elements and frictional elements. At the same time, the bonded elements are transformed into frictional element when the bonded elements are broken during the loading process. Also, the root is abstracted into another non-destructive bonded elements material, which bears the load together. The linear elastic constitutive model is used for root and bonded elements, and the double-hardening model is used for friction elements. Considering the influence of freeze–thaw cycles, the extended binary model is derived here. Finally, the experimental results show that the predicted results of this model are in good agreement with the experimental results, and the new model can relatively well simulate the strain softening and volumetric dilatancy phenomena.

## Introduction

Recently, a large number of infrastructure projects such as railways, highways, airports and water conservancy hubs have been built in areas with seasonal and permanent frozen soils. With the rise of temperature, slopes in permafrost degradation areas and seasonal permafrost areas may be affected by freeze–thaw action, which may lead to their unique freeze–thaw collapse^1^. Slope protection by vegetation is to maintain the stability of rock and soil slope, reduce slope erosion, prevent soil and water loss, and protect the ecological environment by using the principle of vegetation drainage and soil fixation. Meanwhile, it also has the advantages of low cost and superior economy^2^. Therefore, it is necessary to study the influence of freeze–thaw cycle and root system on soil mechanical properties.

Freeze–thaw cycles can lead to significant changes in soil engineering properties. Many scholars’^3–11^ studies about fine grained soils (clay and silt) show that: freeze–thaw cycles will affect soil structure, density, porosity, water redistribution, and the strength, compressibility and pore water pressure. Freeze–thaw cycles can also cause changes in soil microstructure, resulting in changes in soil properties in many ways, such as permeability changes, often up to several levels of magnitude^12^. Although many scholars have made fruitful achievements in the research of frozen soil mechanics in recent years, the results are not uniform. In terms of stress–strain relationship, Alkire et al.^13^ and Ono et al.^14^ respectively conducted experiments on reshaped silty soil and reshaped clay, and the stress–strain curve of soil undergoing a freeze–thaw cycle was above that of unfreeze-thaw soil, indicating that freeze–thaw action enhanced soil's resistance to shear stress. On the contrary, tests conducted by Graham et al.^15^ show that the soil compression curve after freeze–thaw is located below unbroken soil without freeze–thaw cycles. The test results of Wang et al.^16^ on Qinghai-Tibet clay show that, at low confining pressure, the stress–strain relationship of samples after freeze–thaw cycles gradually changes from strain-softening type to strain-hardening type, that is, the breakage mode of samples changes from brittle breakage to plastic breakage due to freeze–thaw action, but if at high confining pressure, the strain hardening was observed before and after freeze–thaw. The results of freeze–thaw cycles effect on soil strength are different. Broms^17^, Leroueil^18^ and Qin et al.^19^ found that soil strength decreased after freeze–thaw cycles. Yong^20^ found that the strength of soil increased after freeze–thaw cycles. However, Bondarenko et al.^21^ and Swan et al.^22^ found that freeze–thaw cycles had little influence on soil strength, and the strength was basically unchanged before and after freeze–thaw cycles. At present, the different mechanical behaviors of soil under freeze–thaw action may be closely related to the test methods used as well as the type and initial state of soil mass. Due to the complex changes of soil physical properties after freeze–thaw cycles, the study on the influence of freeze–thaw cycles on soil mechanical properties and its mechanism is not enough, and no unified conclusion and theory has been formed.

The soil fixation mechanism of plant roots is mainly reflected in three aspects: the anchorage effect of deep roots, the reinforcement effect of shallow roots, and the reduction of slope pore water pressure^23–25^. It is generally believed that the tensile properties of roots have the greatest influence on the mechanical properties of soil containing roots. Some scholars have made in-depth studies on the factors affecting the tensile properties of plant roots, for example, Genet^26^ and Tosi et al.^27^ studied the tensile strength of roots of different trees and shrubs in the Mediterranean region, and showed that there were great differences in the tensile strength of roots of different tree species. The shear stress on the soil is converted into the pull on the roots through the surface friction of the roots^28^. In order to quantify the effect of roots on soil, Wu and Waldron^29,30^ believed that soil reinforcement by roots was mainly reflected in the improvement of soil shear strength, and proposed the Wu-Waldron model of soil shear strength, which considers the influence of plant roots mechanical properties. This model has few parameters and is easy to understand. Most subsequent studies are based on this model to revise and improve. Pollen^31^ argues that one of the assumptions of the Wu-Waldron model (simultaneous and transient fracture of all roots) can overestimate the reinforcement effect of roots on soil, and proposes the Fiber Bundle Model that considers progressive root breakage, and a more reasonable root cohesion is obtained. But this model is affected by the change of root density. Schwarz^32^ proposes the Root Bundle Model, assuming that the friction between the root and loam varies linearly from 0.1 to 10 kPa with the soil volume moisture content. In recent years, studies on atmosphere-vegetation-soil coupling have been developed^33,34^ and Ng et al.^35^ proposed a new model of coupled migration of groundwater seepage and surface runoff considering the influence of plant root shape.

Although many previous scholars have studied freeze–thaw cycles and rooted soil respectively, few experimental studies and theoretical models have taken these two factors into account. In order to explore this issue, the paper firstly studied the mechanical properties of rooted soil under freeze–thaw cycles, and analyzed the influences of confining pressure, freeze–thaw cycle and root system. Then based on the binary medium model^36–45^, the paper extends it to three-phase mixed materials, derives the extended model of binary media, and considers the influence of freeze–thaw cycle. Finally, the validity and applicability of the model are verified by experimental results.

## Test material and method

### Test material

**Figure 1:**
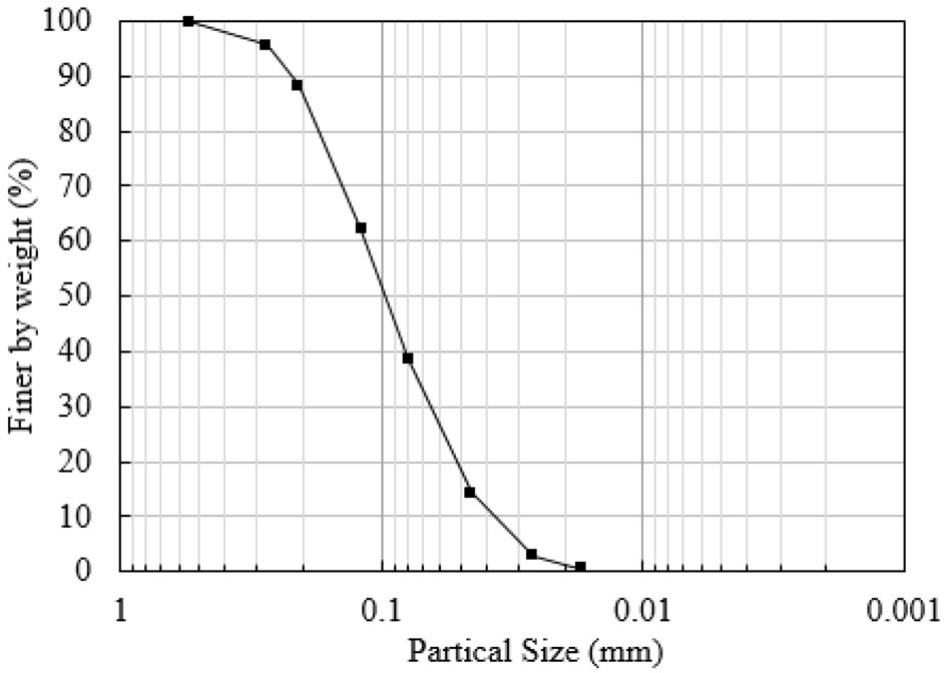
The grain distribution curve of tested soils.

The test soil samples were taken from Hailuogou, Sichuan Province, China at an altitude of about 3000 m, and the depth of the soil samples was within 0.5–1.0 m below the surface. The grain distribution curve of the test soil sample is shown in Fig. 1, and the physical properties are shown in Table 1. According to the classification standard of soil, the soil belongs to silty sand. According to the coefficient of inhomogeneity and the coefficient of curvature, the soil samples were judged to be well graded. The root system of Sorbus was selected. The length of the root was about 5 cm, the diameter of the root was about 5 mm, the weight of each root was about 1.4 g, the mass ratio was 0.292% (dry bulk density), the volume ratio was 0.262%, and the shear area ratio was 0.254%.

**Table 1:** Physical parameters of test soil samples.

Specific gravity (Gs)	Dry density (g/cm^3^)	Void ratio (e)	Natural moisture content (%)	Coefficient uniformity	Coefficient of curvature
2.6	1.28	1.03	21.8%	5.56	1.28

### Sample preparation

**Figure 2:**
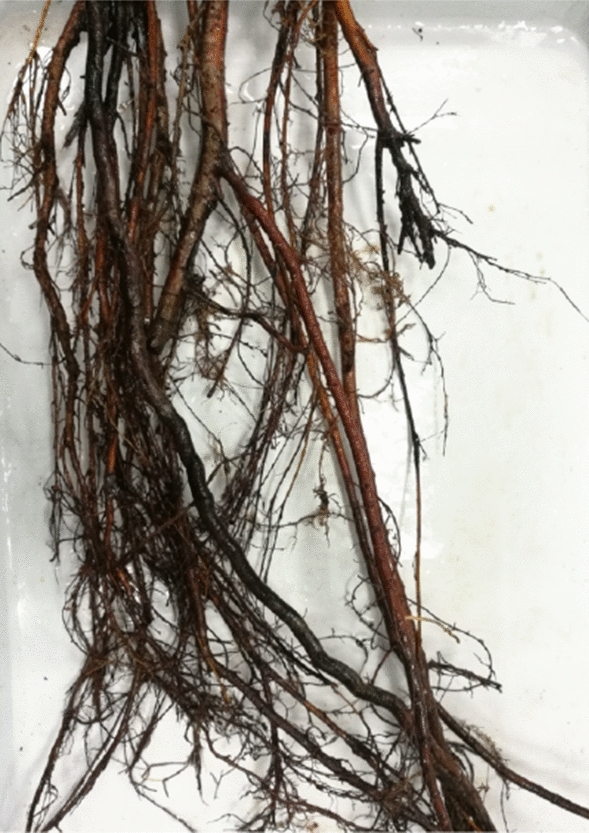
Sorbus root system.

**Figure 3:**
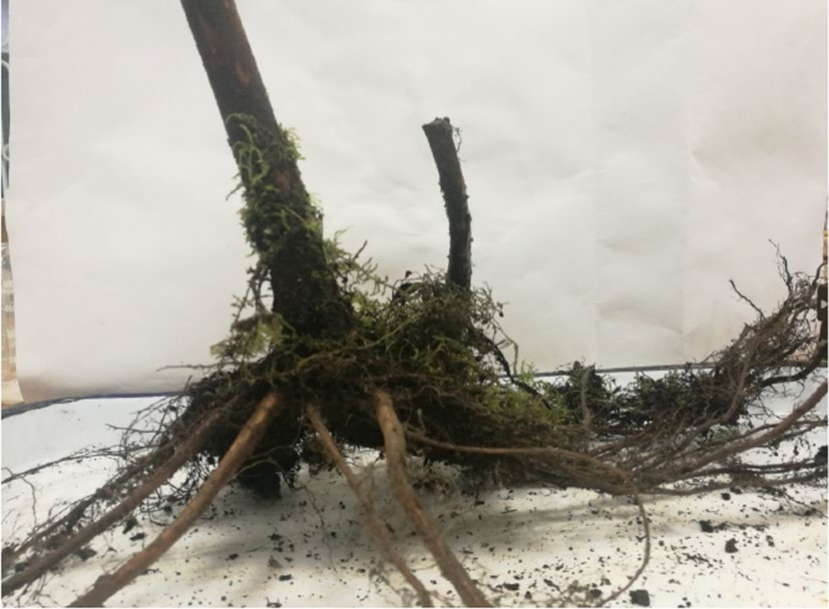
Natural root state of *Sorbus chinensis.*

**Figure 4:**
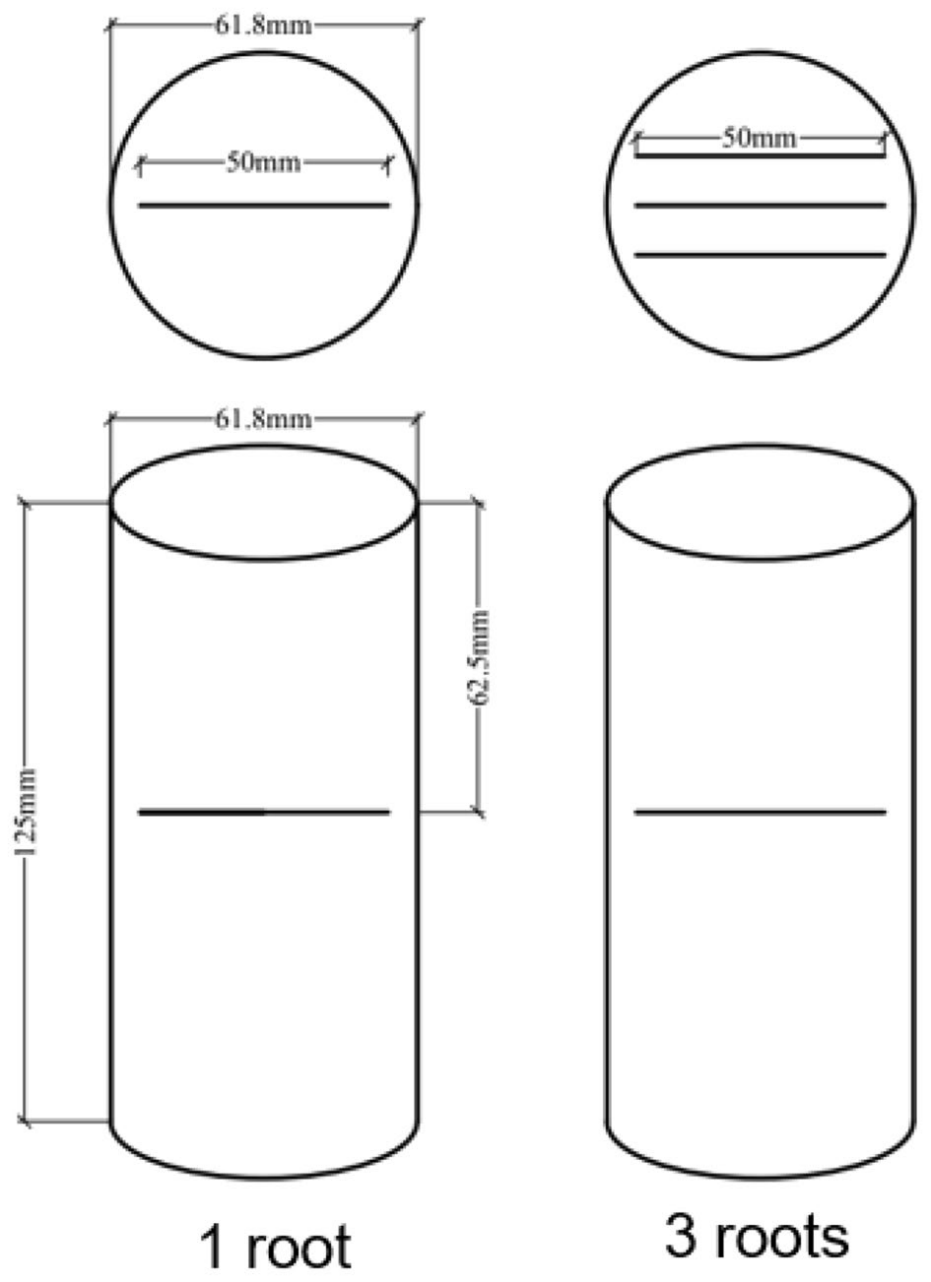
Root layout diagram.

After retrieving the test soil, let it air dry naturally, and pass through a 2 mm sieve after grinding. The soil were mixed evenly at the dry density of 1.28 g/cm^3^, and then packed into the sample preparation cylinder and compacted in four layers, with a size of 61.8 mm in diameter and 125 mm in height. For the samples containing roots, live Sorbus roots were selected, as shown in Fig. 2, then cut out part of the root diameter for about 5 mm and the straight part for 5 cm, and placed them in the middle of the second and the third layer of soil during sample preparation. Since Sorbus roots is generally buried shallowly in the natural production process and grows horizontally, as shown in Fig. 3. Therefore, the root system of the sample was arranged horizontally and placed in the middle of the sample, as shown in Fig. 4.

**Figure 5:**
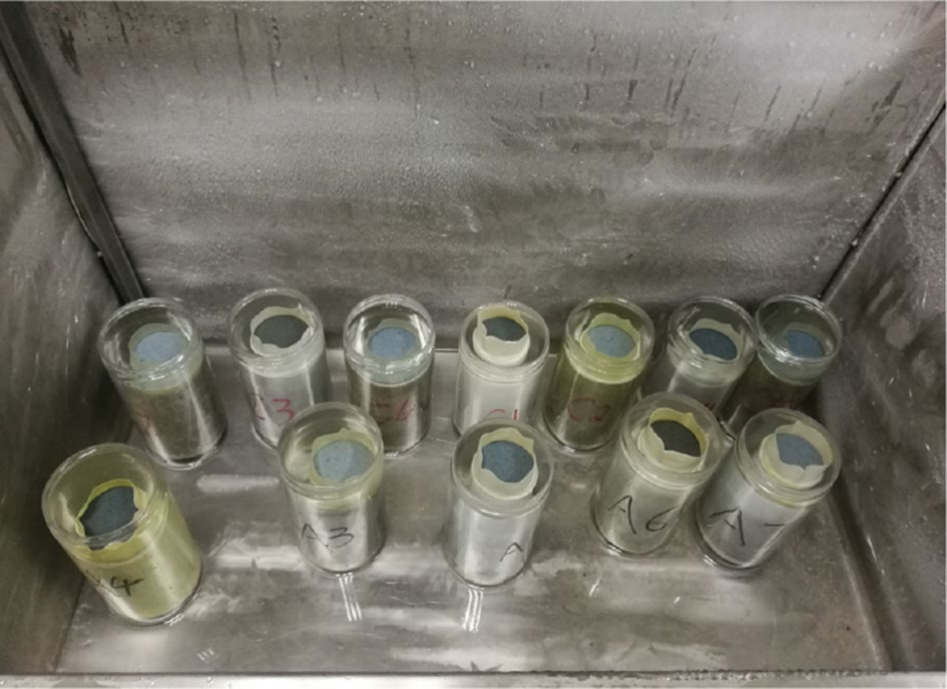
Samples subjected to freeze–thaw cycles.

After the sample is prepared, it is vacuumized for 2 h and then water is released to immerse the sample in hydraulic saturation for more than 12 h. Then take out the saturated sample and cover it with rubber film and permeable stone as soon as possible. Rubber rings are attached to the positions of the permeable stone at both the upper and lower ends to prevent the sample from deforming too much in the process of freeze–thaw cycles. Then, triaxial test is carried out immediately for samples that do not need freeze–thaw cycle. For samples subjected to freeze–thaw cycles, the sample is quickly placed in water to remove air bubbles, and transferred in water to a transparent plastic tank without a lid. The transparent tank with samples was numbered and put into the automatic low-temperature freeze–thaw machine to start the freeze–thaw cycle, as shown Fig. 5. In the process of freeze–thaw cycle, the sample can be replenished by permeable stones at both ends. A freeze–thaw cycle consists of lowering room temperature to – 15 °C for 12 h and then rising to 20 °C for 12 h.

### Test method

After the set number of freeze–thaw cycles, the sample was taken out and installed on the triaxial tester to perform consolidated drained triaxial test at room temperature. The axial loading speed was 0.1 mm/min and the confining pressure was 25, 50, 100, 200 kPa. If the axial deformation is up to 15%, the sample will be considered damaged, the test would stop. The specific test scheme is shown in Table 2, and N in the figures and tables in this paper represents the number of freeze–thaw cycles, and r represents the number of roots contained. The soil after the test is pulverized and reconstituted again, which is called remolded soil, and the sample can be prepared under rootless and non-freeze–thaw conditions for triaxial test, which is used for the determination of some parameters.

**Table 2:** Test plan table.

Test type	Confining pressure/kPa	Number of freeze–thaw cycles	Root number
Consolidated and drained test (CD)	25	0	0
	50	1	1
	100	5	3
	200	15	–

### Ethical approval

Since this study did not recruit any human and/or animal subjects, this section does not apply.

### Consent to participate

Since this study did not recruit any human subjects, this section does not apply.

## Experimental analysis of mechanical properties of rooted soil under freeze–thaw cycles

**Figure 6:**
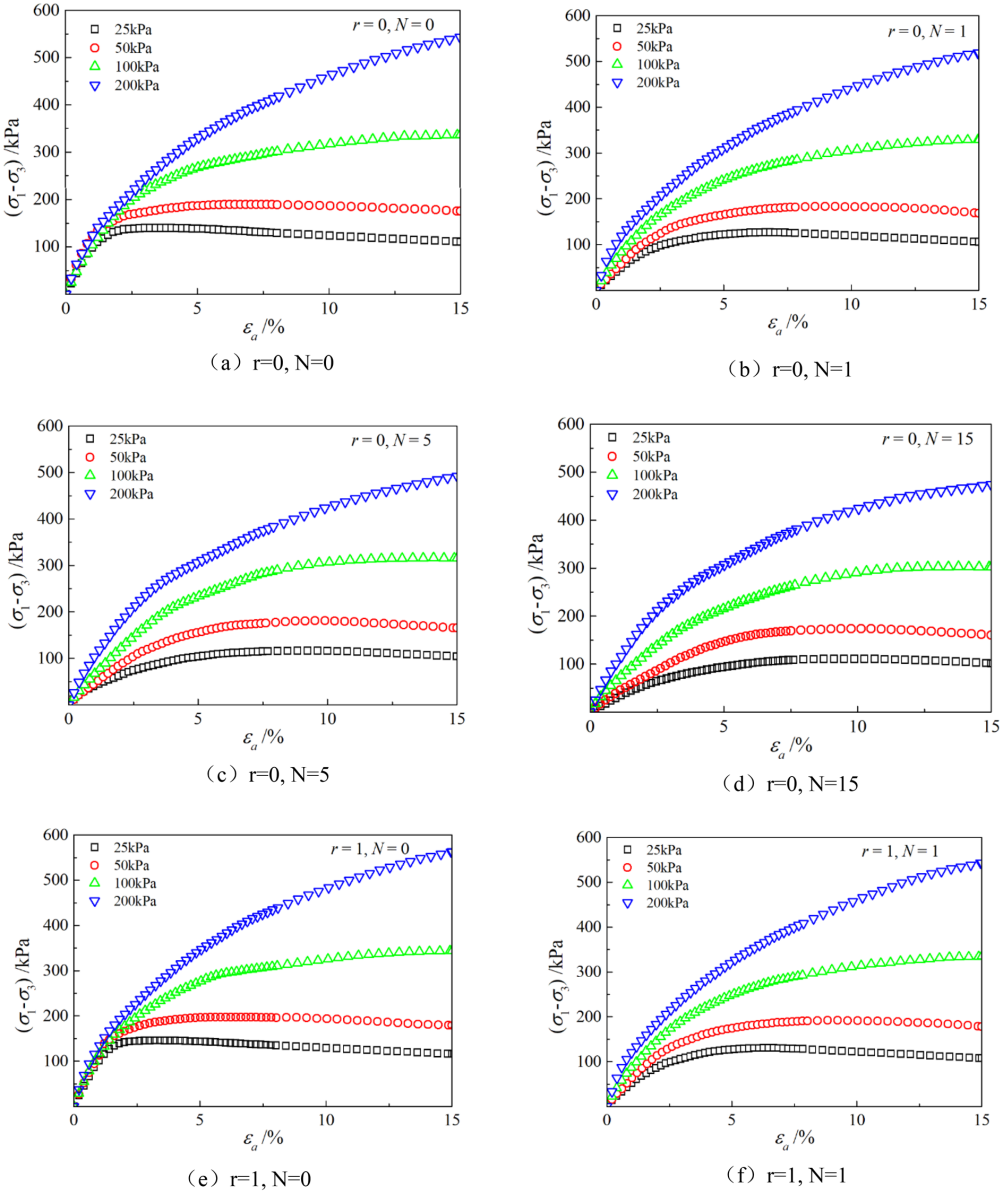

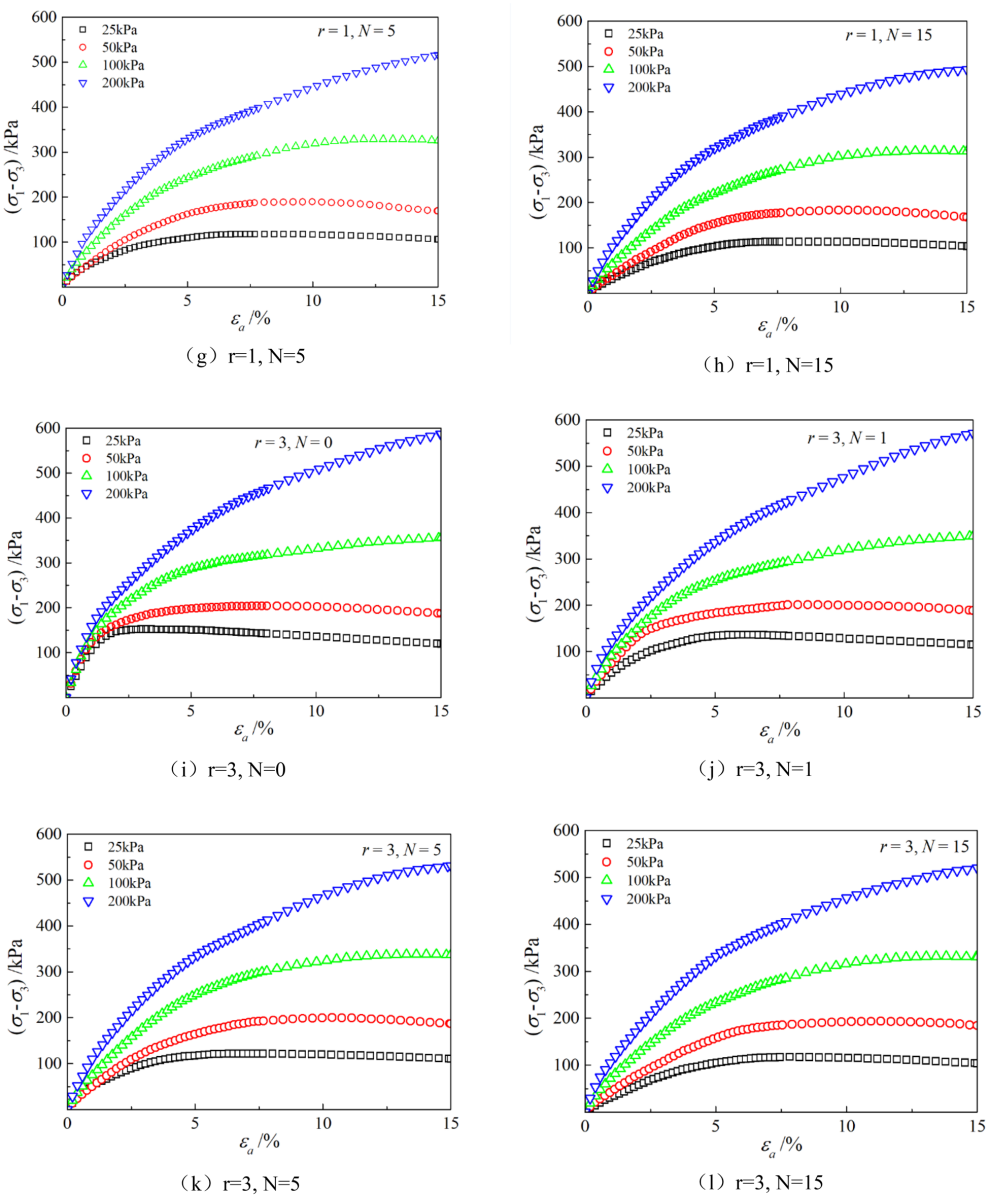
Relations between axial strain and deviatoric stress under different confining pressures.

**Figure 7:**
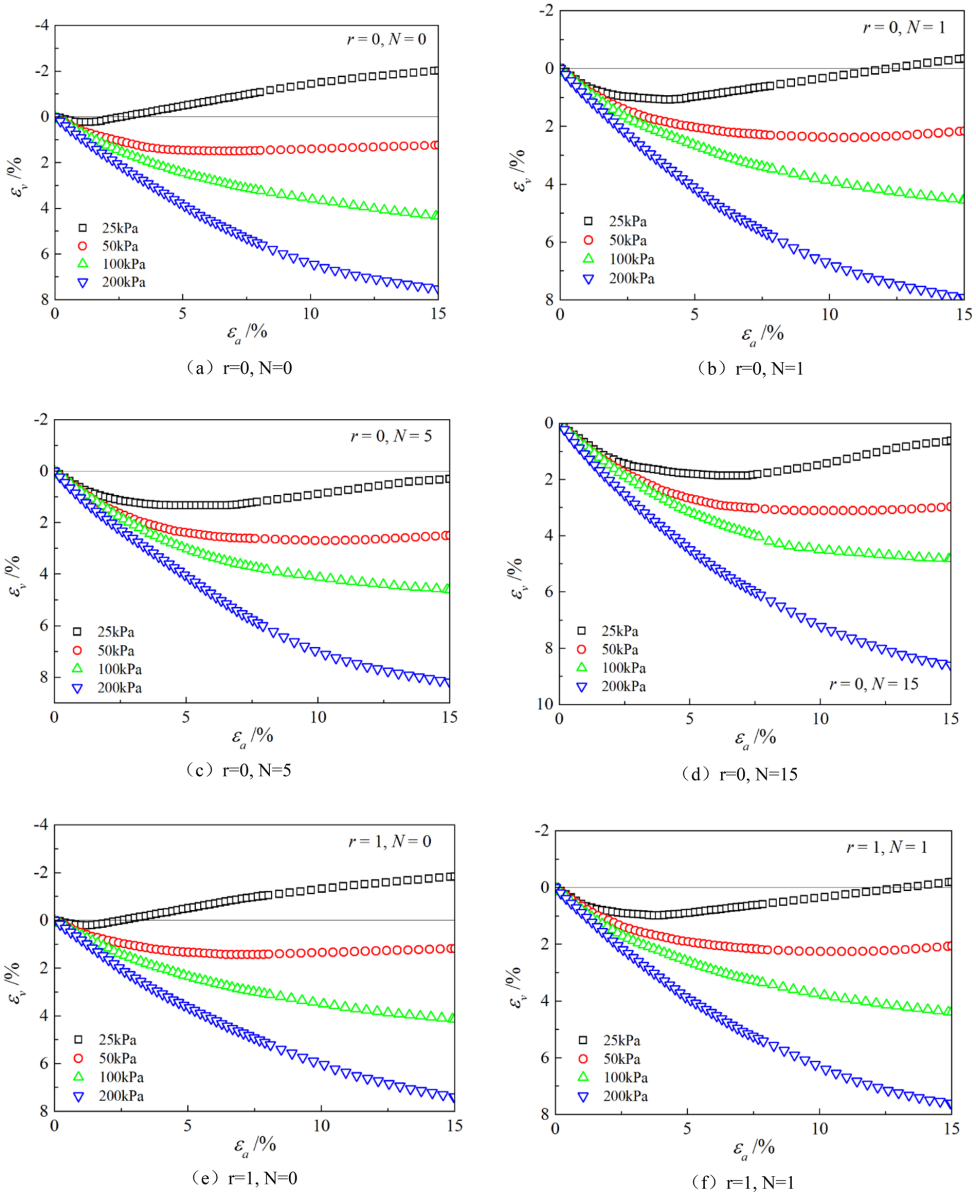

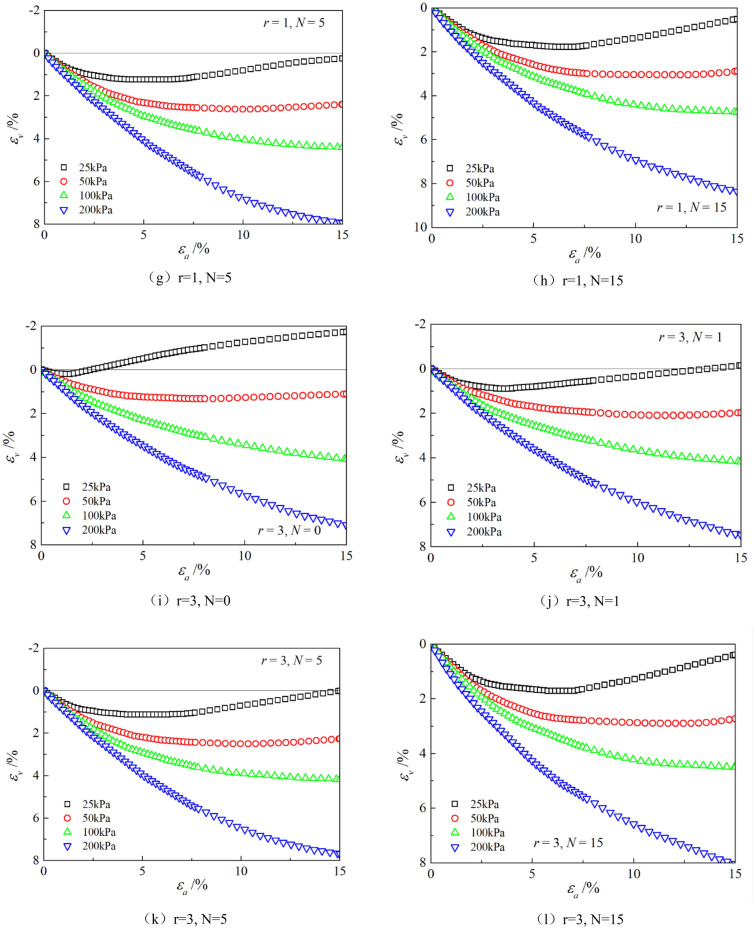
Relations between axial strain and volume strain under different confining pressures.

**Figure 8:**
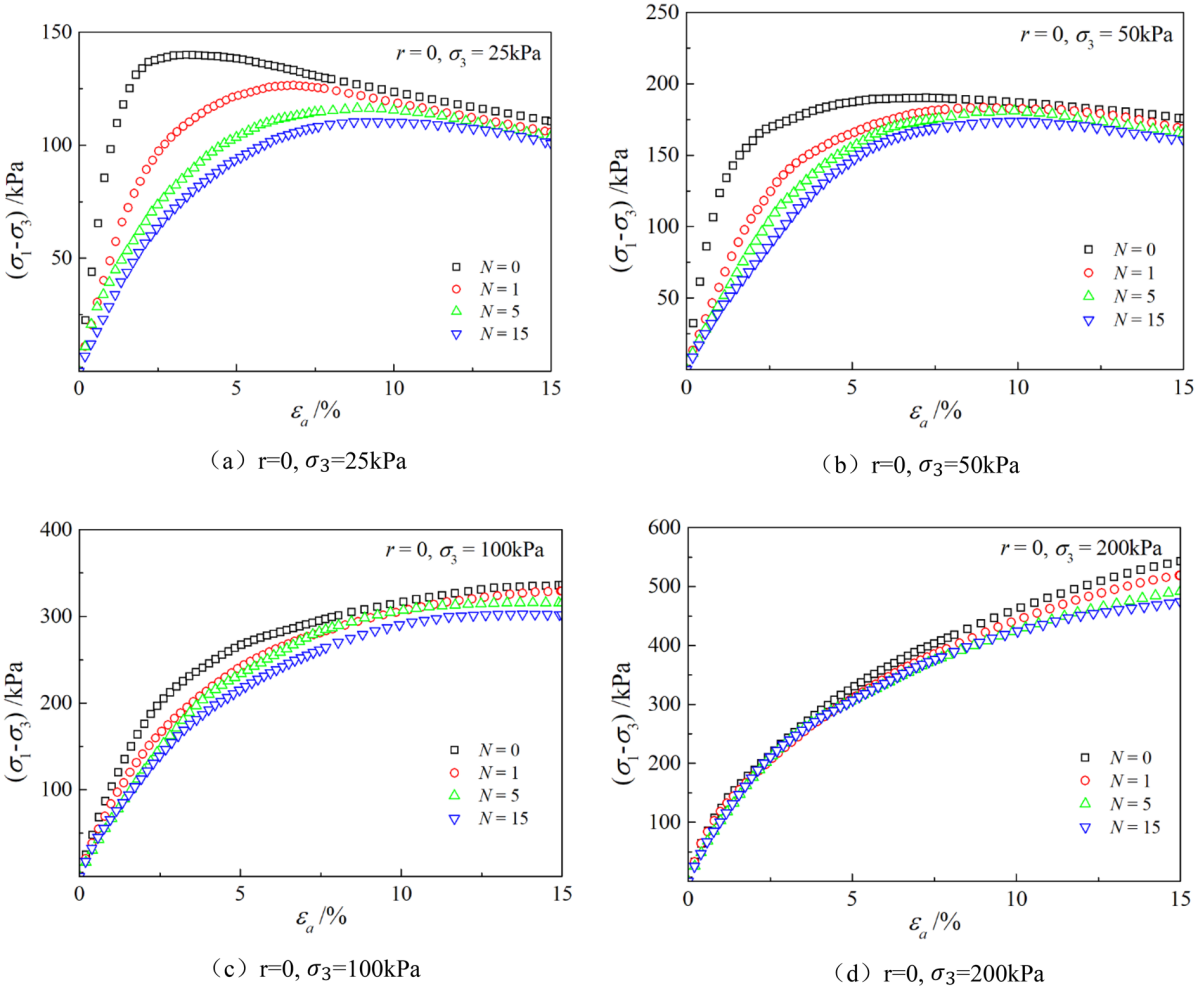
Relations between axial strain and deviatoric stress with different freeze–thaw cycles.

**Figure 9:**
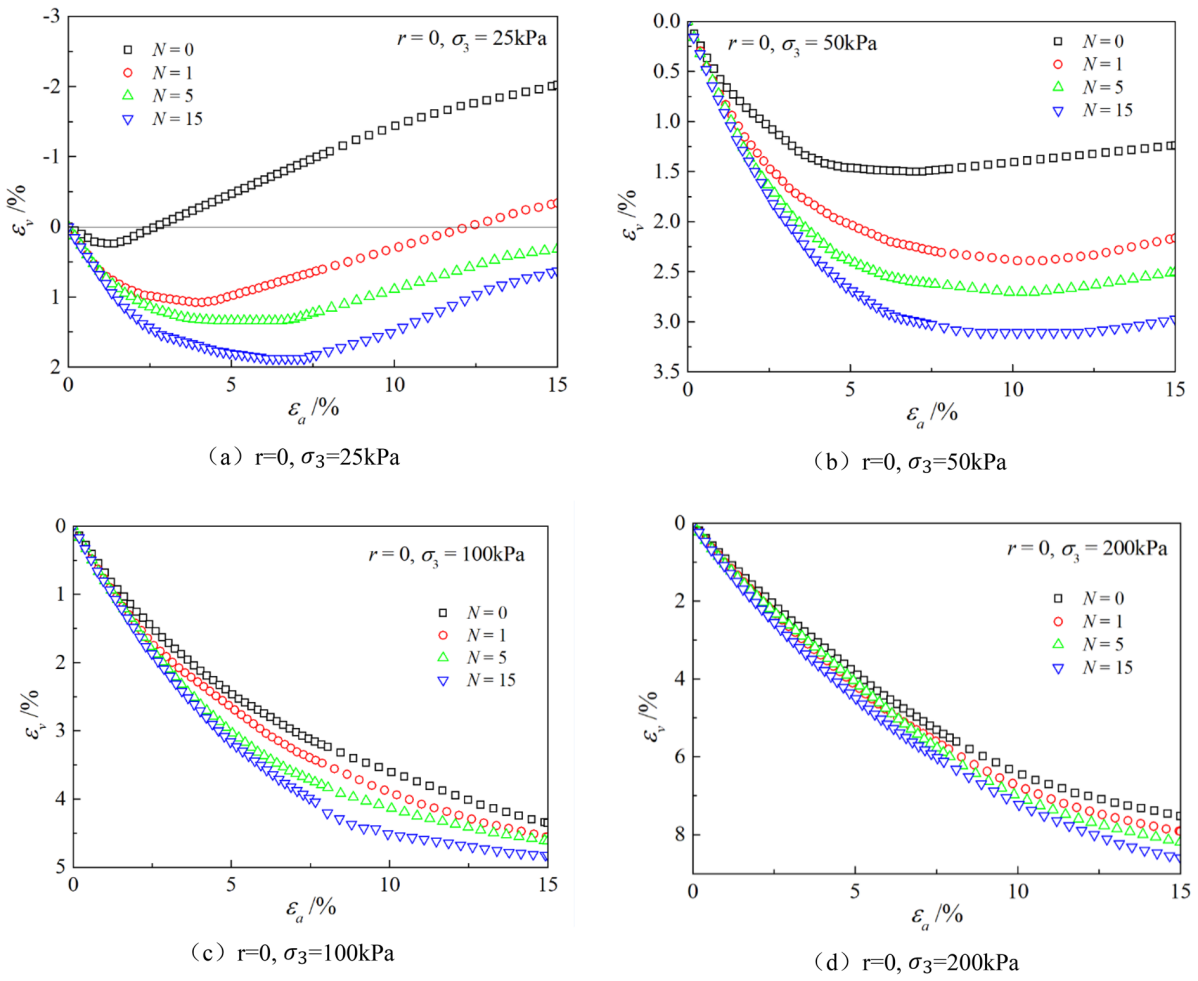
Relations between axial strain and volume strain with different freeze–thaw cycles.

**Figure 10:**
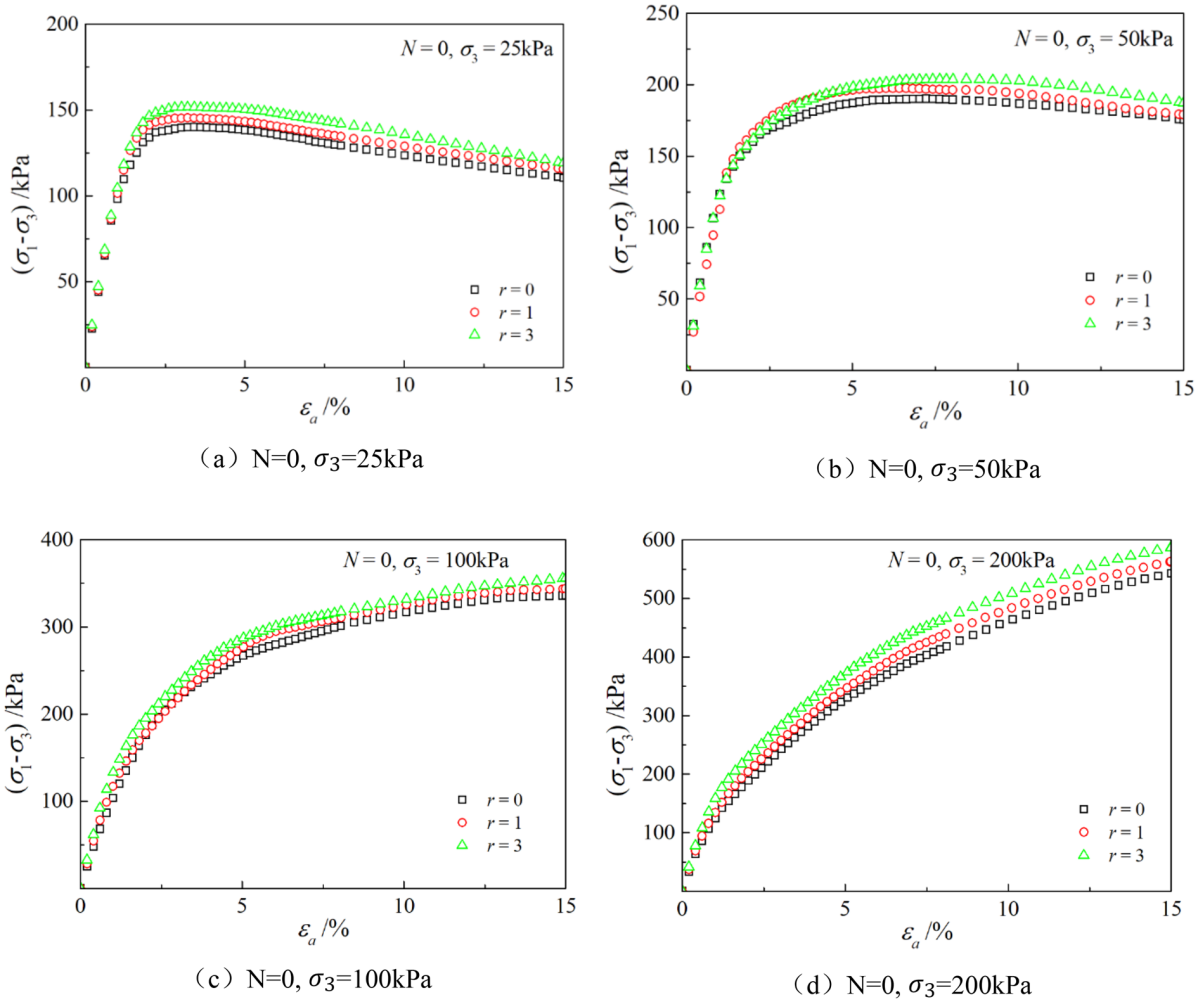
Relationships between axial strain and deviatoric stress with different root number.

**Figure 11:**
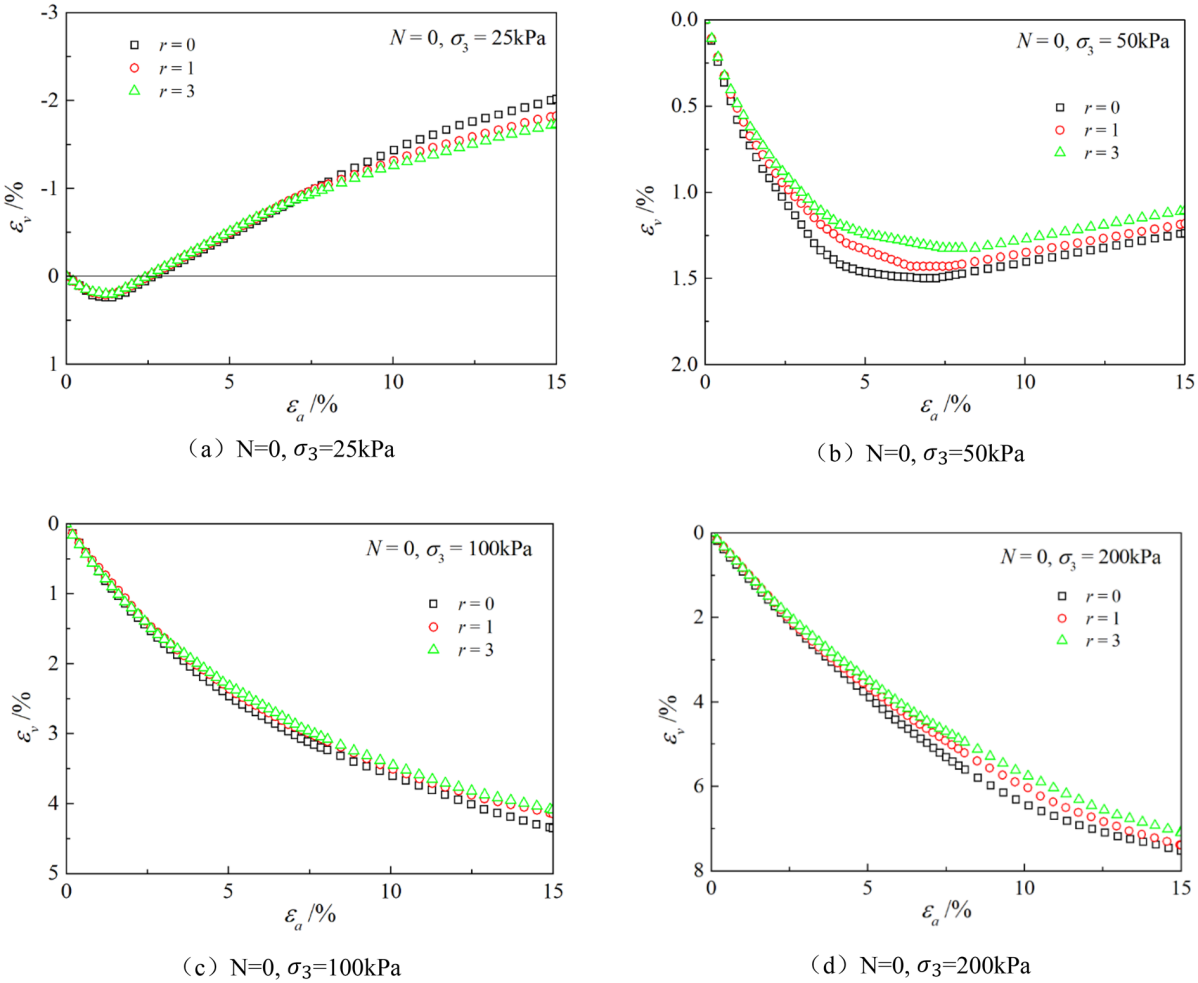
Relationships between axial strain and volume strain with different root number.

Figures 6, 7, 8, 9, 10, 11 respectively describe the relationship curves of deviatoric stress, volumetric strain and axial strain under different confining pressures, different freeze–thaw cycles and different root number. ${\varepsilon }_{a}$ in the figure is for axial strain, ${\varepsilon }_{v}$ is for volumetric strain,${\sigma }_{1}-{\sigma }_{3}$ is for deviatoric stress. It can be seen from the figure that the soil containing roots is greatly affected by confining pressure and the number of freeze–thaw cycles, and the volume ration of roots to sample has strengthening effect on the sample to some degree.

### Influence of confining pressure

**Figure 12:**
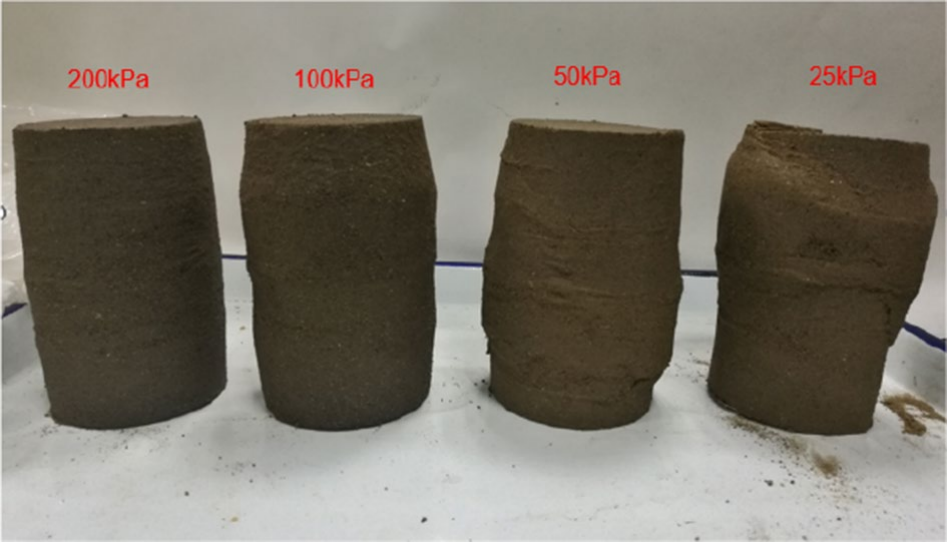
Breakage forms of samples under different confining pressures.

Figures 6 and 7 show that the stress–strain curves of samples are significantly affected by confining pressure. The specimen exhibits strain softening and volumetric dilatancy under low confining pressure. When the confining pressure is 25 kPa, the deviatoric stress of the sample decreases rapidly after reaching the peak strength, and then gradually flattens out, and finally gradually approaches the residual strength. The volumetric strain of the sample expands after a short shrinkage, and the volumetric strain gradually becomes negative (that is, exceeds the initial volume of the sample), and continues to expand. At the confining pressure of 50 kPa, strain softening and volumetric dilatancy still occur, but the phenomenon is weakened. When the deviatoric stress reaches its peak, the strength decreases slowly and finally approaches the residual strength gradually. The volumetric strain of the sample is also the first bulk shrinkage, but the volume shrinkage is larger, and then the bulk expansion is slow, and the volume expansion is relatively small, and the negative bulk change will not be generated eventually. With the increase of confining pressure, the phenomenon of strain softening and swelling disappear gradually. Under high confining pressure, the mechanical properties of the samples are constant strain hardening and volume shrinkage. As the confining pressure increases, the breakage mode of the sample changes from shear breakage to swelling breakage, as shown in Fig. 12. The strength of the sample increases with the increase of confining pressure.

The mechanical properties of rooted soil with respect to confining pressure variation are consistent with those of typical sandy soils, as explained in the article. This is because during the consolidation process, the confining pressure damages the bonding and structural integrity between particles, and the higher the confining pressure, the greater the degree of disruption. At low confining pressures, the bonding and structural integrity between soil particles remain relatively intact during consolidation. When the specimen is sheared, the external load is mainly borne by the bonding elements, and the interlocking between particles in the weak zones of stress concentration is completely destroyed, resulting in strain softening and shear failure. At high confining pressures, the bonding and structural integrity between soil particles undergo varying degrees of damage during consolidation. Some of the bonding elements have transformed into frictional elements, and the external load is mainly borne by the frictional elements. During the shear process, the resistance to sliding and friction between particles predominantly come into play.

### Influence of the number of freeze–thaw cycles

As can be seen from Figs. 8 and 9, with the increase of the number of freeze–thaw cycles, both stress and volumetric strain curves are located below, which means that the deviatoric stress decreases while the volumetric strain increases. At low confining pressure, with the increase of freeze–thaw cycles, the phenomenon of strain softening and volume expansion will be weaker, the deviatoric stress will decrease more gently after reaching the peak, the volume shrinkage will be larger, and the volume dilatancy will be more gentle. At the confining pressure of 25 kPa, the volume change will not reach negative value after the freeze–thaw cycles have reached 5 times. Under high confining pressure, with the increase of freeze–thaw cycles, the bearing capacity of the sample decreases, which is manifested as the deviatoric stress decreases gradually and the volume shrinkage value increases gradually. In general, with the increase of the number of freeze–thaw cycles, the mechanical properties and strength of the samples decreased gradually.

This is because the soil before freeze–thaw is structural and there is cementation among the particles, while in the freeze–thaw cycle, the soil particles bear the frost heaving force. In the freeze–thaw cycle, this bonding between the particles is destroyed, and the sample changes from a relatively dense state to a relatively loose state (‘tight sand’ becomes ‘loose sand’). In the original soil, there existed structural interlocking and bonding between soil particles. During the freezing process, the original bonding structure is disrupted due to the significant frost heave forces acting on the soil particles. However, the generated ice crystals bond the soil particles together, providing new and stronger bonding effects. This is why frozen soil often exhibits increased strength. During the thawing process, the ice crystals transition from a solid to a liquid state, causing the bonding effects of the ice crystals to disappear. After a complete freeze–thaw cycle, a portion of the soil particles undergo damage, transitioning from bonding elements to frictional elements, leading to a decrease in the mechanical properties and bearing capacity of the samples. As the number of freeze–thaw cycles increases, the proportion and degree of soil particle damage increase, resulting in a greater reduction in the mechanical properties and bearing capacity of the samples.

### Effect of volume ration of roots to sample

As can be seen from Figs. 10 and 11, with the increase of volume ration of roots to sample, the deviatoric stress increases, the deformation decreases, and the strength gradually increases. On one hand, According to the homogenization theory of composite materials, plant roots have a certain strengthening effect on the mechanical properties of the samples. The modulus and strength of plant roots in the samples are higher than that of soil, and the bearing capacity of the samples with roots is slightly higher than that without roots. On the other hand, the combination of roots and soil allows them to leverage their respective advantages, with roots providing tensile strength and soil providing compressive strength. When subjected to external loads, both the soil and roots undergo deformation. Due to the significant difference in their deformation moduli, there will be relative displacement or a tendency for relative displacement between the soil and roots. Stress within the soil redistributes, resulting in surface frictional forces between the roots and soil particles. This leads to tensile forces in the roots, which, in turn, hinder soil deformation through frictional resistance, thereby enhancing the overall strength of the soil. But the rising level is not high, because the root system occupies a small proportion of the volume. In actual soil mass, the strength of soil mass is improved to some extent due to the reinforcement effect of shallow roots and the anchorage effect of deep roots, which can be used as a safety reserve for design.

### Strength

When the stress–strain curve is strain softening, the peak value is taken as the shear strength of the sample. When the stress–strain curve is continuous hardening, the corresponding deviatoric stress of and the axial strain is 15% is the shear strength of the sample. The shear strength of all samples is summarized in Tables 3, 4, 5. It can be seen from the table that the strength of the sample increases with the increase of confining pressure (it is also obtained by Tian et al.^46^), decreases with the increase of freeze–thaw cycles, and increases with the increase of the number of roots contained. Comparing specimens with 1, 5, and 15 freeze–thaw cycles to samples without freeze–thaw cycles, the average reduction in strength is 4.66%, 9.08%, and 12.11%, respectively. It is because (1) with the increase of confining pressure, the soil becomes more compact and the shear modulus is larger; (2) In the process of freeze–thaw cycle, the soil structure is gradually destroyed; and (3) the root has reinforcing effect on the soil.

**Table 3:** Summary of shear strength of samples without root (unit: kPa).

Number of freeze–thaw cycles	25 kPa	50 kPa	100 kPa	200 kPa
0	140.00	190.12	335.90	542.69
1	126.56	183.30	329.12	518.79
5	116.22	180.95	315.29	492.14
15	110.33	173.89	302.74	473.90

**Table 4:** Summary of shear strength of samples with one root (unit: kPa).

Number of freeze–thaw cycles	25 kPa	50 kPa	100 kPa	200 kPa
0	145.41	197.96	343.60	563.02
1	130.11	191.97	334.41	542.99
5	117.46	189.50	325.87	516.47
15	113.27	183.51	314.11	493.30

**Table 5:** Summary of shear strength of samples with three roots (unit: kPa).

Number of freeze–thaw cycles	25 kPa	50 kPa	100 kPa	200 kPa
0	151.71	203.81	355.90	587.00
1	135.71	201.18	348.99	570.82
5	120.79	200.03	337.99	531.09
15	117.09	193.36	331.88	519.54

## Extended binary medium model of rooted soil

### Formulation of the constitutive model

**Figure 13:**
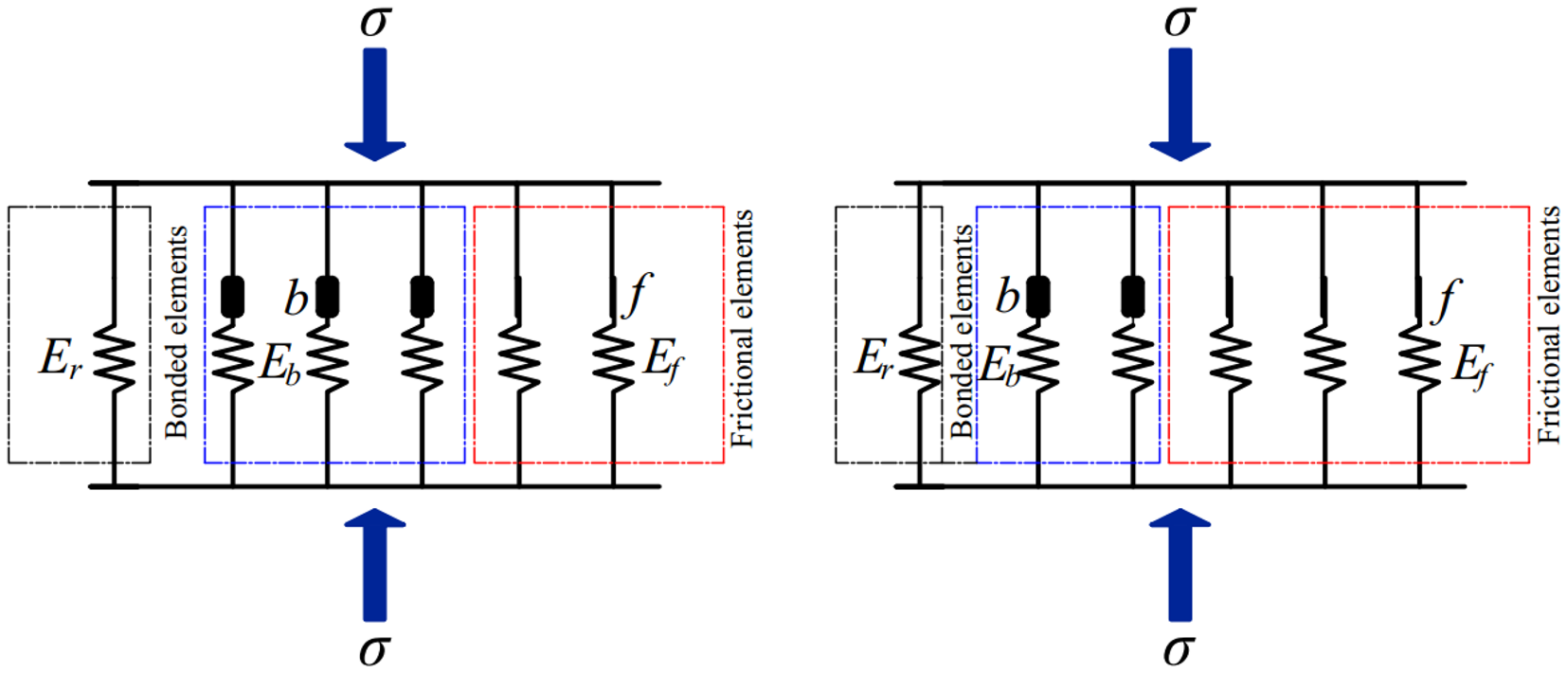
Schematic diagram of the model.

The traditional binary medium model abstracts the soil as a mixture composed of structural blocks and weak zones (called bonded elements and frictional elements, respectively). The load is shared by the bonded elements and frictional elements that will be damaged and transformed. During the loading process, the bonded element is gradually destroyed and transformed into frictional element. Rooted soil is regarded as a heterogeneous material composed of soil and roots. Soil can be regarded as the component of damaged transformed bonded element and friction element, while root can be regarded as another similar bonded elements material that will not be damaged. It is assumed that the strength and modulus of the root are large enough to prevent the root from being damaged during the loading process. The schematic diagram of the model is shown in Fig. 13.

Take a representative volume element (RVE), and the stress and strain on the bonded element, frictional element and root are respectively:

1 $${\sigma }_{ij}^{b}=\frac{1}{{V}^{b}}\int {\sigma }_{ij}^{loc}d{V}^{b}$$

2 $${\varepsilon }_{ij}^{b}=\frac{1}{{V}^{b}}\int {\varepsilon }_{ij}^{loc}d{V}^{b}$$

3 $${\sigma }_{ij}^{f}=\frac{1}{{V}^{f}}\int {\sigma }_{ij}^{loc}d{V}^{f}$$

4 $${\varepsilon }_{ij}^{f}=\frac{1}{{V}^{f}}\int {\varepsilon }_{ij}^{loc}d{V}^{f}$$

5 $${\sigma }_{ij}^{r}=\frac{1}{{V}^{r}}\int {\sigma }_{ij}^{loc}d{V}^{r}$$

6 $${\varepsilon }_{ij}^{r}=\frac{1}{{V}^{r}}\int {\varepsilon }_{ij}^{loc}d{V}^{r}$$

where ${\sigma }_{ij}^{loc}$ and ${\varepsilon }_{ij}^{loc}$ are the local microscopic stress and strain in the RVE. For the RVE, we have

7 $$V={V}^{b}+{V}^{f}+{V}^{r}$$

Based on the homogenization theory of heterogeneous media, the following formula can be derived:

8 $${\sigma }_{ij}=\frac{1}{V}\int {\sigma }_{ij}^{loc}d\mathrm{V}$$

9 $${\varepsilon }_{ij}=\frac{1}{V}\int {\varepsilon }_{ij}^{loc}d\mathrm{V}$$

Substituting Eqs. (1)–(7) into (8) and (9), we get:

10 $${\sigma }_{ij}=\frac{{V}^{b}}{V}{\sigma }_{ij}^{b}+\frac{{V}^{f}}{V}{\sigma }_{ij}^{f}+\frac{{V}^{r}}{V}{\sigma }_{ij}^{r}$$

11 $${\varepsilon }_{ij}=\frac{{V}^{b}}{V}{\varepsilon }_{ij}^{b}+\frac{{V}^{f}}{V}{\varepsilon }_{ij}^{f}+\frac{{V}^{r}}{V}{\varepsilon }_{ij}^{r}$$

Definition ${\lambda }_{v}^{f}=\frac{{V}^{f}}{V}$ represents volume breakage ratio; ${\lambda }_{v}^{r}=\frac{{V}^{r}}{V}$ represents the volume ration of roots to sample. *λ* represents the stress sharing ratio, and Eqs. (10) and (11) are transformed into:

12 $${\sigma }_{ij}=\left(1-{\lambda }^{r}-{\lambda }^{f}\right){\sigma }_{ij}^{b}+{\lambda }^{f}{\sigma }_{ij}^{f}+{\lambda }^{r}{\sigma }_{ij}^{r}$$

13 $${\varepsilon }_{ij}=\left(1-{\lambda }^{r}-{\lambda }^{f}\right){\varepsilon }_{ij}^{b}+{\lambda }^{f}{\varepsilon }_{ij}^{f}+{\lambda }^{r}{\varepsilon }_{ij}^{r}$$

Even if the stress–strain relationship of the bonded element and root system adopts linear elastic constitutive relationship, the stress–strain relationship of the frictional element is still nonlinear elastic–plastic. Therefore, the previous formula cannot simulate the actual stress–strain relationship, so it is necessary to use the linear method by replacing the elastic method to conduct full differentiation of Eqs. (12) and (13), and change them into incremental expressions, as follows:

14 $$d{\sigma }_{ij}=\left(1-{\lambda }^{r}-{\lambda }^{f}\right){d\sigma }_{ij}^{b}+{\lambda }^{f}d{\sigma }_{ij}^{f}+{\lambda }^{r}{d\sigma }_{ij}^{r}+d{\lambda }^{f}\left({\sigma }_{ij}^{f0}-{\sigma }_{ij}^{b0}\right)+d{\lambda }^{r}\left({\sigma }_{ij}^{r0}-{\sigma }_{ij}^{b0}\right)$$

15 $${d\varepsilon }_{ij}=\left(1-{\lambda }^{r}-{\lambda }^{f}\right)d{\varepsilon }_{ij}^{b}+{\lambda }^{f}d{\varepsilon }_{ij}^{f}+{\lambda }^{r}d{\varepsilon }_{ij}^{r}+d{\lambda }^{f}\left({\varepsilon }_{ij}^{f0}-{\varepsilon }_{ij}^{b0}\right)+d{\lambda }^{r}\left({\varepsilon }_{ij}^{r0}-{\varepsilon }_{ij}^{b0}\right)$$

It can be seen from Eqs. (14) and (15) that the average stress–strain increment of the representative element is composed of four parts, including the influence of bonded element, frictional element, root and breakage ratio.

### Two-parameter binary medium model

The stress is decomposed into the spherical stress and deviatoric stress, i.e. mean stress and generalized shear stress. The strain is decomposed into the volume strain and shear strain.

16 $${\sigma }_{m}=\frac{1}{3}{\sigma }_{kk}$$

17 $${\sigma }_{s}=\sqrt{\frac{3}{2}\left({\sigma }_{ij}-\frac{1}{3}{\delta }_{ij}{\sigma }_{kk}\right)\left({\sigma }_{ij}-\frac{1}{3}{\delta }_{ij}{\sigma }_{kk}\right)}$$

18 $${\varepsilon }_{v}={\varepsilon }_{kk}$$

19 $${\varepsilon }_{s}=\sqrt{\frac{2}{3}\left({\varepsilon }_{ij}-\frac{1}{3}{\delta }_{ij}{\varepsilon }_{kk}\right)\left({\varepsilon }_{ij}-\frac{1}{3}{\delta }_{ij}{\varepsilon }_{kk}\right)}$$

Since it is assumed that the root will not be destroyed in the loading process and ${\lambda }^{r}$ is considered to be constant, and ${d\lambda }^{r}=0$, then:

20 $${d\sigma }_{m}=\left(1-{\lambda }_{v}^{r0}-{\lambda }_{v}^{f0}\right)d{\sigma }_{m}^{b}+{\lambda }_{v}^{f0}{d\sigma }_{m}^{f}+{\lambda }_{v}^{r0}d{\sigma }_{m}^{r}+d{\lambda }_{v}^{f}\left({\sigma }_{m}^{f0}-{\sigma }_{m}^{b0}\right)$$

21 $${d\varepsilon }_{v}=\left(1-{\lambda }_{v}^{r0}-{\lambda }_{v}^{f0}\right){d\varepsilon }_{v}^{b}+{\lambda }_{v}^{f0}{d\varepsilon }_{v}^{f}+{\lambda }_{v}^{r0}d{\varepsilon }_{v}^{r}+d{\lambda }_{v}^{f}\left({\varepsilon }_{v}^{f0}-{\varepsilon }_{v}^{b0}\right)$$

22 $${d\sigma }_{s}=\left(1-{\lambda }_{s}^{r0}-{\lambda }_{s}^{f0}\right)d{\sigma }_{s}^{b}+{\lambda }_{s}^{f0}{d\sigma }_{s}^{f}+{\lambda }_{s}^{r0}d{\sigma }_{s}^{r}+d{\lambda }_{s}^{f}\left({\sigma }_{s}^{f0}-{\sigma }_{s}^{b0}\right)$$

23 $$d{\varepsilon }_{s}=\left(1-{\lambda }_{s}^{r0}-{\lambda }_{s}^{f0}\right){d\varepsilon }_{s}^{b}+{\lambda }_{s}^{f0}{d\varepsilon }_{s}^{f}+{\lambda }_{s}^{r0}d{\varepsilon }_{s}^{r}+d{\lambda }_{s}^{f}\left({\varepsilon }_{s}^{f0}-{\varepsilon }_{s}^{b0}\right)$$

Similarly, ${\lambda }_{s}^{f}$ is defined as the area breakage ratio during cutting process, and ${\lambda }_{s}^{r}$ is the area ratio of roots to sample.

From Eqs. (20) to (23), we can get:

24 $${d\sigma }_{m}^{f}=\frac{1}{{\lambda }_{v}^{f0}}\left[{d\sigma }_{m}-\left(1-{\lambda }_{v}^{r0}-{\lambda }_{v}^{f0}\right)d{\sigma }_{m}^{b}-{\lambda }_{v}^{r0}d{\sigma }_{m}^{r}-d{\lambda }_{v}^{f}\left({\sigma }_{m}^{f0}-{\sigma }_{m}^{b0}\right)\right]$$

25 $${d\sigma }_{s}^{f}=\frac{1}{{\lambda }_{s}^{f0}}\left[{d\sigma }_{s}-\left(1-{\lambda }_{s}^{r0}-{\lambda }_{s}^{f0}\right)d{\sigma }_{s}^{b}-{\lambda }_{s}^{r0}d{\sigma }_{s}^{r}-d{\lambda }_{s}^{f}\left({\sigma }_{s}^{f0}-{\sigma }_{s}^{b0}\right)\right]$$

26 $${d\varepsilon }_{v}^{f}=\frac{1}{{\lambda }_{v}^{f0}}\left[{d\varepsilon }_{v}-\left(1-{\lambda }_{v}^{r0}-{\lambda }_{v}^{f0}\right){d\varepsilon }_{v}^{b}-{\lambda }_{v}^{r0}d{\varepsilon }_{v}^{r}-d{\lambda }_{v}^{f}\left({\varepsilon }_{v}^{f0}-{\varepsilon }_{v}^{b0}\right)\right]$$

27 $${d\varepsilon }_{s}^{f}=\frac{1}{{\lambda }_{s}^{f0}}\left[d{\varepsilon }_{s}-\left(1-{\lambda }_{s}^{r0}-{\lambda }_{s}^{f0}\right){d\varepsilon }_{s}^{b}-{\lambda }_{s}^{r0}d{\varepsilon }_{s}^{r}-d{\lambda }_{s}^{f}\left({\varepsilon }_{s}^{f0}-{\varepsilon }_{s}^{b0}\right)\right]$$

The constitutive relation of the bonded elements and root is assumed to be linear elasticity:

28 $${d\varepsilon }_{v}^{b}=\frac{1}{{K}^{b}}d{\sigma }_{m}^{b}$$

29 $${d\varepsilon }_{s}^{b}=\frac{1}{3{G}^{b}}d{\sigma }_{s}^{b}$$

30 $${d\varepsilon }_{v}^{r}=\frac{1}{{K}^{r}}d{\sigma }_{m}^{r}$$

31 $${d\varepsilon }_{s}^{r}=\frac{1}{3{G}^{r}}d{\sigma }_{s}^{r}$$

The frictional element adopts the nonlinear elastic–plastic constitutive, and the volume strain and shear strain are the coupling relations between the spherical stress and deviator stress:

32 $${d\varepsilon }_{v}^{f}={K}_{v}^{f}{d\sigma }_{m}^{f}+{K}_{s}^{f}{d\sigma }_{s}^{f}$$

33 $${d\varepsilon }_{s}^{f}={G}_{v}^{f}{d\sigma }_{m}^{f}+{G}_{s}^{f}{d\sigma }_{s}^{f}$$

Due to the non-uniformity of deformation of cement element, frictional elements and root in the RVE, local stress concentration coefficient is introduced to represent the relationship between local stress of bonded element, root and average stress of the REV.

34 $${\sigma }_{m}^{b}={C}_{m}^{b}{\sigma }_{m}$$

35 $${\sigma }_{s}^{b}={C}_{s}^{b}{\sigma }_{s}$$

36 $${\sigma }_{m}^{r}={C}_{m}^{r}{\sigma }_{m}$$

37 $${\sigma }_{s}^{r}={C}_{s}^{r}{\sigma }_{s}$$

The incremental forms of Eqs. (34) to (37) are:

38 $${d\sigma }_{m}^{b}={{\sigma }_{m}^{0}d{C}_{m}^{b}+C}_{m}^{b0}d{\sigma }_{m}={B}_{m}^{b}d{\sigma }_{m}$$

39 $${d\sigma }_{s}^{b}={{\sigma }_{s}^{0}d{C}_{S}^{b}+C}_{s}^{b0}d{\sigma }_{s}={B}_{S}^{b}d{\sigma }_{s}$$

40 $${d\sigma }_{m}^{r}={{\sigma }_{m}^{0}d{C}_{m}^{r}+C}_{m}^{r0}d{\sigma }_{m}={B}_{m}^{r}d{\sigma }_{m}$$

41 $${d\sigma }_{s}^{r}={{\sigma }_{s}^{0}d{C}_{S}^{r}+C}_{s}^{r0}d{\sigma }_{s}={B}_{s}^{r}d{\sigma }_{s}$$

42 $${B}_{m}^{b}={C}_{m}^{b0}+{\frac{\partial {C}_{m}^{b}}{\partial {\sigma }_{m}}}\sigma_{m}^{0}$$

43 $${B}_{s}^{b}={C}_{s}^{b0}+{\frac{\partial {C}_{s}^{b}}{\partial {\sigma }_{s}}}\sigma_{s}^{0}$$

44 $${B}_{m}^{r}={C}_{m}^{r0}+{\frac{\partial {C}_{m}^{r}}{\partial {\sigma }_{m}}}\sigma_{m}^{0}$$

45 $${B}_{s}^{b}={C}_{s}^{b0}+{\frac{\partial {C}_{s}^{r}}{\partial {\sigma }_{s}}}\sigma_{s}^{0}$$

Then, two parameters, volume breakage ratio and area breakage ratio, were introduced to describe the breakage characteristics of bonded elements and frictional elements. The expression is:

46 $${\lambda }_{v}^{f}={f}_{1}({\sigma }_{m},N)$$

47 $${\lambda }_{s}^{f}={f}_{2}({\sigma }_{s},N)$$

where ${f}_{1}$ is the function of the spherical stress and the number of freeze–thaw cycles, ${f}_{2}$ is the function of deviatoric stress and the number of freeze–thaw cycles.

The total differentiation of Eqs. (46) and (47) can be obtained as follows:

48 $${d\lambda }_{v}^{f}=\frac{\partial {f}_{1}}{\partial {\sigma }_{m}}{d\sigma }_{m}+\frac{\partial {f}_{1}}{\partial N}dN$$

49 $${d\lambda }_{s}^{f}=\frac{\partial {f}_{2}}{\partial {\sigma }_{s}}{d\sigma }_{s}+\frac{\partial {f}_{2}}{\partial N}dN$$

According to Eqs. (12), (13), (34–37), we can get:

50 $${\sigma }_{m}^{f0}-{\sigma }_{m}^{b0}=\frac{\left(1-{C}_{m}^{b0}\right)}{{\lambda }_{v}^{f0}}{\sigma }_{m}^{0}+\frac{{\lambda }_{v}^{r0}}{{\lambda }_{v}^{f0}}\left({C}_{m}^{b0}-{C}_{m}^{r0}\right){\sigma }_{m}^{0}$$

51 $${\sigma }_{s}^{f0}-{\sigma }_{s}^{b0}=\frac{\left(1-{C}_{s}^{b0}\right)}{{\lambda }_{s}^{f0}}{\sigma }_{s}^{0}+\frac{{\lambda }_{s}^{r0}}{{\lambda }_{s}^{f0}}\left({C}_{s}^{b0}-{C}_{s}^{r0}\right){\sigma }_{s}^{0}$$

52 $${\varepsilon }_{v}^{f0}-{\varepsilon }_{v}^{b0}=\frac{1}{{\lambda }_{v}^{f0}}\left[\left({\varepsilon }_{v}^{0}{-\varepsilon }_{v}^{b0}\right)+{\lambda }_{v}^{r0}\left({\varepsilon }_{v}^{b0}-{\varepsilon }_{v}^{r0}\right)\right]$$

53 $${\varepsilon }_{s}^{f0}-{\varepsilon }_{s}^{b0}=\frac{1}{{\lambda }_{s}^{f0}}\left[\left({\varepsilon }_{s}^{0}{-\varepsilon }_{s}^{b0}\right)+{\lambda }_{s}^{r0}\left({\varepsilon }_{s}^{b0}-{\varepsilon }_{s}^{r0}\right)\right]$$

By combining Eqs. (20)–(49), we can finally get:

54 $${d\varepsilon }_{v}={A}_{1}d{\sigma }_{m}+{B}_{1}d{\sigma }_{s}+{C}_{1}d\mathrm{N}$$

55 $${d\varepsilon }_{s}={A}_{2}d{\sigma }_{m}+{B}_{2}d{\sigma }_{s}+{C}_{2}d\mathrm{N}$$

Among them:

56 $${A}_{1} =\left[{K}_{v}^{f}+\left(1-{\lambda }_{v}^{r0}-{\lambda }_{v}^{f0}\right)\left(\frac{1}{{K}^{b}}-{K}_{v}^{f}\right){B}_{m}^{b} +{\lambda }_{v}^{r0}\left(\frac{1}{{K}^{r}}-{K}_{v}^{f}\right){B}_{m}^{r}\right]+\frac{1}{{\lambda }_{v}^{f0}}\left\{\left[\left({\varepsilon }_{v}^{0}{-\varepsilon }_{v}^{b0}\right)+{\lambda }_{v}^{r0}\left({\varepsilon }_{v}^{b0}-{\varepsilon }_{v}^{r0}\right)\right]-{K}_{v}^{f}\left[\left(1-{C}_{m}^{b0}\right){\sigma }_{m}^{0}+{\lambda }_{v}^{r0}\left({C}_{m}^{b0}-{C}_{m}^{r0}\right){\sigma }_{m}^{0}\right]\right\}\frac{\partial {f}_{1}}{\partial {\sigma }_{m}}$$

57 $${B}_{1}=\frac{{\lambda }_{v}^{f0}}{{\lambda }_{s}^{f0}}{K}_{s}^{f}\left\{1-\left(1-{\lambda }_{s}^{r0}-{\lambda }_{s}^{f0}\right){B}_{S}^{b}-{\lambda }_{s}^{r0}{B}_{s}^{r}-\left[\frac{\left(1-{C}_{s}^{b0}\right)}{{\lambda }_{s}^{f0}}{\sigma }_{s}^{0}+\frac{{\lambda }_{s}^{r0}}{{\lambda }_{s}^{f0}}\left({C}_{s}^{b0}-{C}_{s}^{r0}\right){\sigma }_{s}^{0}\right]\frac{\partial {f}_{2}}{\partial {\sigma }_{s}}\right\}$$

58 $${C}_{1}=\frac{1}{{\lambda }_{v}^{f0}}\left\{\left[\left({\varepsilon }_{v}^{0}{-\varepsilon }_{v}^{b0}\right)+{\lambda }_{v}^{r0}\left({\varepsilon }_{v}^{b0}-{\varepsilon }_{v}^{r0}\right)\right]-{K}_{v}^{f}\left[\left(1-{C}_{m}^{b0}\right){\sigma }_{m}^{0}+{\lambda }_{v}^{r0}\left({C}_{m}^{b0}-{C}_{m}^{r0}\right){\sigma }_{m}^{0}\right]\right\}\frac{\partial {f}_{1}}{\partial N}-\frac{{\lambda }_{v}^{f0}}{{\left({\lambda }_{s}^{f0}\right)}^{2}}{K}_{s}^{f}\left[{\left(1-{C}_{s}^{b0}\right)}\sigma_{s}^{0}+{\lambda }_{s}^{r0}\left({C}_{s}^{b0}-{C}_{s}^{r0}\right){\sigma }_{s}^{0}\right]\frac{\partial {f}_{2}}{\partial N}$$

59 $${A}_{2}=\frac{{\lambda }_{s}^{f0}}{{\lambda }_{v}^{f0}}{G}_{v}^{f}\left[1-\left(1-{\lambda }_{v}^{r0}-{\lambda }_{v}^{f0}\right){B}_{m}^{b}-{\lambda }_{v}^{r0}{B}_{m}^{r}\right]-\frac{{\lambda }_{s}^{f0}}{{\left({\lambda }_{v}^{f0}\right)}^{2}}{G}_{v}^{f}\left[\left(1-{C}_{m}^{b0}\right){\sigma }_{m}^{0}+{\lambda }_{v}^{r0}\left({C}_{m}^{b0}-{C}_{m}^{r0}\right){\sigma }_{m}^{0}\right]\frac{\partial {f}_{1}}{\partial {\sigma }_{m}}$$

60 $${B}_{2}=\left[{G}_{s}^{f}+\left(1-{\lambda }_{s}^{r0}-{\lambda }_{s}^{f0}\right)\left(\frac{1}{3{G}^{b}}-{G}_{s}^{f}\right) {B}_{S}^{b}+{\lambda }_{s}^{r0}\left(\frac{1}{3{G}^{r}}-{G}_{s}^{f}\right){B}_{s}^{r}\right]+\frac{1}{{\lambda }_{s}^{f0}}\left\{\left({\varepsilon }_{s}^{0}{-\varepsilon }_{s}^{b0}\right)+{\lambda }_{s}^{r0}\left({\varepsilon }_{s}^{b0}-{\varepsilon }_{s}^{r0}\right)-\left[\left(1-{C}_{s}^{b0}\right){\sigma }_{s}^{0}+{\lambda }_{s}^{r0}\left({C}_{s}^{b0}-{C}_{s}^{r0}\right){\sigma }_{s}^{0}\right]{G}_{s}^{f}\right\}\frac{\partial {f}_{2}}{\partial {\sigma }_{s}}$$

61 $${C}_{2}=\frac{1}{{\lambda }_{s}^{f0}}\left\{\left({\varepsilon }_{s}^{0}{-\varepsilon }_{s}^{b0}\right)+{\lambda }_{s}^{r0}\left({\varepsilon }_{s}^{b0}-{\varepsilon }_{s}^{r0}\right)-\left[\left(1-{C}_{s}^{b0}\right){\sigma }_{s}^{0}+{\lambda }_{s}^{r0}\left({C}_{s}^{b0}-{C}_{s}^{r0}\right){\sigma }_{s}^{0}\right]{G}_{s}^{f}\right\}\frac{\partial {f}_{2}}{\partial N}-\frac{{\lambda }_{s}^{f0}}{{\left({\lambda }_{v}^{f0}\right)}^{2}}{G}_{v}^{f}\left[\left(1-{C}_{m}^{b0}\right){\sigma }_{m}^{0}+{\lambda }_{v}^{r0}\left({C}_{m}^{b0}-{C}_{m}^{r0}\right){\sigma }_{m}^{0}\right]\frac{\partial {f}_{1}}{\partial N}$$

When N = 0, there is no dN and it becomes a binary medium model containing root soil.

When ${\lambda }^{r}={\lambda }_{v}^{r}={\lambda }_{s}^{r}=0$, it becomes a rootless freeze–thaw cycle binary medium model, and the coefficient is taken as ${A}_{1}^{{{\prime}}}$, ${B}_{1}^{{{\prime}}}$, ${C}_{1}^{{{\prime}}}$, ${A}_{2}^{{{\prime}}}$, ${B}_{2}^{{{\prime}}}$, ${C}_{2}^{{{\prime}}}$.

62 $${A}_{1}^{{{\prime}}}={K}_{v}^{f}+\left(1-{\lambda }_{v}^{f0}\right)\left(\frac{1}{{K}^{b}}-{K}_{v}^{f}\right){B}_{m}^{b}+\frac{1}{{\lambda }_{v}^{f0}}\left[\left({\varepsilon }_{v}^{0}{-\varepsilon }_{v}^{b0}\right)-{K}_{v}^{f}\left(1-{C}_{m}^{b0}\right){\sigma }_{m}^{0}\right]\frac{\partial {f}_{1}}{\partial {\sigma }_{m}}$$

63 $${B}_{1}^{{{\prime}}}=\frac{{\lambda }_{v}^{f0}}{{\lambda }_{s}^{f0}}{K}_{s}^{f}\left[1-\left(1-{\lambda }_{s}^{f0}\right){B}_{S}^{b}\right]-\frac{{\lambda }_{v}^{f0}}{{\left({\lambda }_{s}^{f0}\right)}^{2}}{K}_{s}^{f}\left(1-{C}_{s}^{b0}\right){\sigma }_{s}^{0}\frac{\partial {f}_{2}}{\partial {\sigma }_{s}}$$

64 $${C}_{1}^{{{\prime}}}=\frac{1}{{\lambda }_{v}^{f0}}\left[\left({\varepsilon }_{v}^{0}{-\varepsilon }_{v}^{b0}\right)-{K}_{v}^{f}\left(1-{C}_{m}^{b0}\right){\sigma }_{m}^{0}\right]\frac{\partial {f}_{1}}{\partial N}-\frac{{\lambda }_{v}^{f0}}{{\left({\lambda }_{s}^{f0}\right)}^{2}}{K}_{s}^{f}\left(1-{C}_{s}^{b0}\right){\sigma }_{s}^{0}\frac{\partial {f}_{2}}{\partial N}$$

65 $${A}_{2}^{{{\prime}}}=\frac{{\lambda }_{s}^{f0}}{{\lambda }_{v}^{f0}}{G}_{v}^{f}\left[1-\left(1-{\lambda }_{v}^{f0}\right){B}_{m}^{b}\right]-\frac{{\lambda }_{s}^{f0}}{{\left({\lambda }_{v}^{f0}\right)}^{2}}{G}_{v}^{f}\left(1-{C}_{m}^{b0}\right){\sigma }_{m}^{0}\frac{\partial {f}_{1}}{\partial {\sigma }_{m}}$$

66 $${B}_{2}^{{{\prime}}}={G}_{s}^{f}+\left(1-{\lambda }_{s}^{f0}\right)\left(\frac{1}{3{G}^{b}}-{G}_{s}^{f}\right) {B}_{S}^{b}+\frac{1}{{\lambda }_{s}^{f0}}\left[\left({\varepsilon }_{s}^{0}{-\varepsilon }_{s}^{b0}\right)-\left(1-{C}_{s}^{b0}\right){{\sigma }_{s}^{0}G}_{s}^{f}\right]\frac{\partial {f}_{2}}{\partial {\sigma }_{s}}$$

67 $${C}_{2}^{{{\prime}}}=\frac{1}{{\lambda }_{s}^{f0}}\left[\left({\varepsilon }_{s}^{0}{-\varepsilon }_{s}^{b0}\right)-\left(1-{C}_{s}^{b0}\right){{\sigma }_{s}^{0}G}_{s}^{f}\right]\frac{\partial {f}_{2}}{\partial N}-\frac{{\lambda }_{s}^{f0}}{{\left({\lambda }_{v}^{f0}\right)}^{2}}{G}_{v}^{f}\left(1-{C}_{m}^{b0}\right){\sigma }_{m}^{0}\frac{\partial {f}_{1}}{\partial N}$$

When ${\lambda }^{r}={\lambda }_{v}^{r}={\lambda }_{s}^{r}=0$, then N = 0, it is simplified to a general two-parameter binary medium model without dN term, and the coefficient is taken as ${A}_{1}^{{{\prime}}}$, ${B}_{1}^{{{\prime}}}$, ${A}_{2}^{{{\prime}}}$, ${B}_{2}^{{{\prime}}}$.

### Model parameter determination

#### Constitutive relation of the bonded elements

It is assumed that the bonded elements is an ideal elastic-brittle material. Before the stress state reaches the breakage level, it is characteristic of the linear elastic constitutive model. As followed by Hooke’s law, it is presented in Eqs. (28) and (29). ${K}^{b} \; \mathrm{and } \; {G}^{b}$ can be tested by in-situ test or laboratory test of geotechnical materials. For example, ${K}^{b }\mathrm{and }{G}^{b}$ can be identified by the initial slope of curves ${\varepsilon }_{v}-{\sigma }_{m}$ and ${{\varepsilon }_{s}-\sigma }_{s}$ of conventional consolidated drained triaxial test. Or the elastic modulus *E* is approximated by the deformation modulus of 0.2% axial strain, and then from $K=\frac{E}{3(1-2\nu )}$ and $G=\frac{E}{2(1+\nu )}$, it can derive that $\nu$ is the Poisso’s ratio.

#### Constitutive relation of the frictional elements

When the external stress of the bonded elements reaches the breakage level, the cementing bond breaks and the bonded elements transforms into the frictional elements to continue to bear the load. Frictional elements can be considered as isotropic elastoplastic materials. In order to better simulate the strain-softening and volumetric dilatancy phenomena of samples, the double-hardening constitutive model with wide applicability, which is proposed by Shen, is adopted in this paper^47,48^.

According to classical elastoplastic theory, the total strain is composed of elastic strain and plastic strain. Hooke’s law is adopted for elastic strain and plastic theory is adopted for plastic strain. The strain increment of frictional elements can be expressed as:

68 $${d\varepsilon }_{v}^{f}={d\varepsilon }_{v}^{fe}+{d\varepsilon }_{v}^{fp}$$

69 $${d\varepsilon }_{s}^{f}={d\varepsilon }_{s}^{fe}+{d\varepsilon }_{s}^{fp}$$

The elastic stress–strain relationship of frictional elements is:

70 $${d\varepsilon }_{v}^{fe}=\frac{{d\sigma }_{m}^{f}}{{K}^{f}}$$

71 $${d\varepsilon }_{s}^{fe}=\frac{{d\sigma }_{s}^{f}}{{3G}^{f}}$$

The double-hardening constitutive model includes two hardening parameters, plastic volumetric strain and plastic shear strain, and its yield function is:

72 $${f}^{f}=\frac{{\sigma }_{m}^{f}}{1-{({\eta }^{f}/\alpha )}^{n}}-H$$

73 $$\alpha ={\alpha }_{m}\left[1.0-{c}_{1}exp\left({\varepsilon }_{s}^{fp}/{c}_{2}\right)\right]$$

74 $$H={p}_{0}exp\left(\beta {\varepsilon }_{v}^{fp}\right)$$

where:

75 $${\eta }^{f}=\frac{{\sigma }_{s}^{f}}{{\sigma }_{m}^{f}}$$

76 $$\beta =\frac{1+{e}_{0}}{\lambda {\prime}-{\rm K}}$$

In order to better reflect the deformation characteristics of frictional elements, the non-associated flow law is adopted, that is, the yield function and the plastic potential function are the same. The plastic potential function is:

77 $${g}^{f}=\frac{{\sigma }_{m}^{f}}{1-{({\eta }^{f}/\alpha )}^{{n}_{1}}}-H$$

According to the flow rule:

78 $${d\varepsilon }_{v}^{fp}=d\Lambda \frac{\partial {g}^{f}}{\partial {\sigma }_{m}^{f}}$$

79 $${d\varepsilon }_{s}^{fp}=d\Lambda \frac{\partial {g}^{f}}{\partial {\sigma }_{s}^{f}}$$

in which $$d\Lambda >0$$.

The consistency condition is applied Eq. (72), i.e.

80 $$\frac{\partial {f}^{f}}{\partial {\sigma }_{m}^{f}}{d\sigma }_{m}^{f}+\frac{\partial {f}^{f}}{\partial {\sigma }_{s}^{f}}{d\sigma }_{s}^{f}+\frac{\partial {f}^{f}}{\partial \alpha }\frac{\partial \alpha }{\partial {\varepsilon }_{s}^{fp}}d{\varepsilon }_{s}^{fp}+\frac{\partial {f}^{f}}{\partial H}\frac{\partial H}{\partial {\varepsilon }_{v}^{fp}}{d\varepsilon }_{v}^{fp}=0$$

We get:

81 $$d\Lambda =\frac{1}{h}\left(\frac{\partial {f}^{f}}{\partial {\sigma }_{m}^{f}}{d\sigma }_{m}^{f}+\frac{\partial {f}^{f}}{\partial {\sigma }_{s}^{f}}{d\sigma }_{s}^{f}\right)$$

82 $$h=-\frac{\partial {f}^{f}}{\partial H}\frac{\partial H}{\partial {\varepsilon }_{v}^{fp}}\frac{\partial {g}^{f}}{\partial {\sigma }_{m}^{f}}-\frac{\partial {f}^{f}}{\partial \alpha }\frac{\partial \alpha }{\partial {\varepsilon }_{s}^{fp}}\frac{\partial {g}^{f}}{\partial {\sigma }_{s}^{f}}$$

In which:

83 $$\frac{\partial {f}^{f}}{\partial {\sigma }_{m}^{f}}=\frac{1-(1+n){({\eta }^{f}/\alpha )}^{n}}{{\left[1-{({\eta }^{f}/\alpha )}^{n}\right]}^{2}}$$

84 $$\frac{\partial {g}^{f}}{\partial {\sigma }_{m}^{f}}=\frac{1-(1+{n}_{1}){({\eta }^{f}/\alpha )}^{{n}_{1}}}{{\left[1-{({\eta }^{f}/\alpha )}^{{n}_{1}}\right]}^{2}}$$

85 $$\frac{\partial {f}^{f}}{\partial {\sigma }_{s}^{f}}=\frac{n{({\eta }^{f}/\alpha )}^{n-1}}{{\alpha \left[1-{({\eta }^{f}/\alpha )}^{n}\right]}^{2}}$$

86 $$\frac{\partial {g}^{f}}{\partial {\sigma }_{s}^{f}}=\frac{{n}_{1}{({\eta }^{f}/\alpha )}^{{n}_{1}-1}}{{\alpha \left[1-{({\eta }^{f}/\alpha )}^{{n}_{1}}\right]}^{2}}$$

87 $$\frac{\partial {f}^{f}}{\partial H}=-1$$

88 $$\frac{\partial H}{\partial {\varepsilon }_{v}^{fp}}={p}_{0}\beta exp\left(\beta {\varepsilon }_{v}^{fp}\right)$$

89 $$\frac{\partial {f}^{f}}{\partial \alpha }=-\frac{n{\sigma }_{m}^{f}{({\eta }^{f}/\alpha )}^{n}}{\alpha {\left[1-{({\eta }^{f}/\alpha )}^{n}\right]}^{2}}$$

90 $$\frac{\partial \alpha }{\partial {\varepsilon }_{s}^{fp}}=-\frac{{\alpha }_{m}{c}_{1}}{{c}_{2}}exp\left({\varepsilon }_{s}^{fp}/{c}_{2}\right)$$

The plastic volumetric strain and shear strain of frictional elements are:

91 $${d\varepsilon }_{v}^{fp}=\frac{1}{h}\left(\frac{\partial {f}^{f}}{\partial {\sigma }_{m}^{f}}\frac{\partial {g}^{f}}{\partial {\sigma }_{m}^{f}}{d\sigma }_{m}^{f}+\frac{\partial {f}^{f}}{\partial {\sigma }_{s}^{f}}\frac{\partial {g}^{f}}{\partial {\sigma }_{m}^{f}}{d\sigma }_{s}^{f}\right)$$

92 $${d\varepsilon }_{s}^{fp}=\frac{1}{h}\left(\frac{\partial {f}^{f}}{\partial {\sigma }_{m}^{f}}{\frac{\partial {g}^{f}}{\partial {\sigma }_{s}^{f}}d\sigma }_{m}^{f}+\frac{\partial {f}^{f}}{\partial {\sigma }_{s}^{f}}\frac{\partial {g}^{f}}{\partial {\sigma }_{s}^{f}}{d\sigma }_{s}^{f}\right)$$

The total stress–strain formula of frictional elements is as follows:

93 $${d\varepsilon }_{v}^{f}=\frac{{d\sigma }_{m}^{f}}{{K}^{f}}+\frac{1}{h}\left(\frac{\partial {f}^{f}}{\partial {\sigma }_{m}^{f}}\frac{\partial {g}^{f}}{\partial {\sigma }_{m}^{f}}{d\sigma }_{m}^{f}+\frac{\partial {f}^{f}}{\partial {\sigma }_{s}^{f}}\frac{\partial {g}^{f}}{\partial {\sigma }_{m}^{f}}{d\sigma }_{s}^{f}\right)$$

94 $${d\varepsilon }_{s}^{f}=\frac{{d\sigma }_{s}^{f}}{{3G}^{f}}+\frac{1}{h}\left(\frac{\partial {f}^{f}}{\partial {\sigma }_{m}^{f}}{\frac{\partial {g}^{f}}{\partial {\sigma }_{s}^{f}}d}\sigma_{m}^{f}+\frac{\partial {f}^{f}}{\partial {\sigma }_{s}^{f}}\frac{\partial {g}^{f}}{\partial {\sigma }_{s}^{f}}{d\sigma }_{s}^{f}\right)$$

By comparing formula (32) and (33), we can obtain:

95 $${K}_{v}^{f}=\frac{1}{{K}^{f}}+\frac{1}{h}\frac{\partial {f}^{f}}{\partial {\sigma }_{m}^{f}}\frac{\partial {g}^{f}}{\partial {\sigma }_{m}^{f}}$$

96 $${K}_{s}^{f}=\frac{1}{h}\frac{\partial {f}^{f}}{\partial {\sigma }_{s}^{f}}\frac{\partial {g}^{f}}{\partial {\sigma }_{m}^{f}}$$

97 $${G}_{v}^{f}=\frac{1}{h}\frac{\partial {f}^{f}}{\partial {\sigma }_{m}^{f}}\frac{\partial {g}^{f}}{\partial {\sigma }_{s}^{f}}$$

98 $${G}_{s}^{f}=\frac{1}{{3G}^{f}}+\frac{1}{h}\frac{\partial {f}^{f}}{\partial {\sigma }_{s}^{f}}\frac{\partial {g}^{f}}{\partial {\sigma }_{s}^{f}}$$

The samples are all composed of frictional elements after complete damage. It can be approximately considered that the soil after the completion of the first test has been completely destroyed. Therefore, the parameters of frictional elements can be obtained through the consolidated drained triaxial test of the reshaped samples.${K}^{f}$ and ${G}^{f}$ were approximately determined by the initial slope of ${\varepsilon }_{v}^{f}-{\sigma }_{m}^{f}$ and ${\varepsilon }_{s}^{f}-{\sigma }_{s}^{f}$ curves of the reshaped samples. $\lambda {\prime}$ and*κ* were determined by the e-lnp curves of the reconstructed samples. ${\alpha }_{m}$, c_1_, c_2_, n, n_1_ were determined by curve fitting of the triaxial test of the reshaped sample.

#### Constitutive relation of roots

Assuming that the root is also an ideal elastic-brittle material, the linear elastic constitutive model is adopted and Hooke's law is followed, as shown in Eqs. (30) and (31). And the strength and modulus of the root are large enough that the root will not be destroyed before the sample is destroyed. ${K}^{r}$ and ${G}^{r}$ can be measured directly or indirectly through laboratory tests.

#### Structural parameters

##### Breakage ratio

*Λ* is an internal variable, as a structural mesoscopic parameter of soil, and is related to the strength of the bonded elements, confining pressure, external load, stress path, so it can not be measured directly. The breakage ratio caused by spherical stress is volume breakage ratio ${\lambda }_{v}^{f}$, and the breakage ratio caused by deviatoric stress is area breakage ratio ${\lambda }_{s}^{f}$. According to the definition and physical meaning of the breakage ratio, it can be seen that the breakage ratio is an undecreasing function varying in the range of 0 to 1. When there are roots, the breakage ratio is limited to $1-{\lambda }_{v}^{r}$. When the specimen is strain softening, the breakage ratio does not increase after reaching the peak pressure and remains unchanged. Therefore, the definition similar to damage variable can be adopted, Weibull distribution function is used to describe the change rule of breakage ratio, and it is assumed that ${\lambda }_{v}^{f}$ and ${\lambda }_{s}^{f}$ conform to the change rule of the following expression:

99 $${\lambda }_{v}^{f}={f}_{1}\left({\sigma }_{m},N\right)=1-{\lambda }_{v}^{r}-\left(1-{\lambda }_{v}^{r}\right)exp\left\{-{\alpha }_{v}{\left(\frac{{\sigma }_{m}}{{P}_{a}}\right)}^{{\theta }_{v}}-{\beta }_{v}{N}^{{\omega }_{v}}\right\} \;\; \text{when} \;\;{d\sigma }_{m}>0$$

100 $${\lambda }_{v}^{f}={\lambda }_{vmax }^{f} \;\; \text{when} \;\;d{\sigma }_{m}\le 0$$

101 $${\lambda }_{s}^{f}={f}_{2}\left({\sigma }_{s},N\right)=1-{\lambda }_{s}^{r}-\left(1-{\lambda }_{s}^{r}\right)exp\left\{-{\alpha }_{s}{\left(\frac{{\sigma }_{s}}{{P}_{a}}\right)}^{{\theta }_{s}}-{\beta }_{s}{N}^{{\omega }_{s}}\right\} \;\; \text{when} \;\; {d\sigma }_{s}>0$$

102 $${\lambda }_{s}^{f}={\lambda }_{smax }^{f}\;\; \text{when} \;\; d{\sigma }_{m}\le 0$$

in which ${P}_{a}$ is the atmospheric pressure, ${\alpha }_{v}, {\beta }_{v}$, ${\theta }_{v}, {\omega }_{v}$, ${\alpha }_{s}$, ${\beta }_{s}$, ${\theta }_{s}$, ${\omega }_{s}$ is the test parameter, fitted by the triaxial test curve of the sample.

From Eqs. (99) to (102), we can get:

103 $$\frac{\partial {f}_{1}}{\partial {\sigma }_{m}}={\frac{1-{\lambda }_{v}^{r}}{{P}_{a}}}\alpha_{v}{{\theta }_{v}\left(\frac{{\sigma }_{m}}{{P}_{a}}\right)}^{{\theta }_{v}-1}exp\left\{-{\alpha }_{v}{\left(\frac{{\sigma }_{m}}{{P}_{a}}\right)}^{{\theta }_{v}}-{\beta }_{v}{N}^{{\omega }_{v}}\right\} \;\; \mathrm{when } \;\; {d\sigma }_{m}>0$$

104 $$\frac{\partial {f}_{1}}{\partial {\sigma }_{m}}=0, \;\; \mathrm{ when } \;\; d{\sigma }_{s}\le 0$$

105 $$\frac{\partial {f}_{1}}{\partial N}=\left(1-{\lambda }_{v}^{r}\right){\beta }_{v}{{\omega }_{v}N}^{{\omega }_{v}-1}exp\left\{-{\alpha }_{v}{\left(\frac{{\sigma }_{m}}{{P}_{a}}\right)}^{{\theta }_{v}}-{\beta }_{v}{N}^{{\omega }_{v}}\right\}$$

106 $$\frac{\partial {f}_{2}}{\partial {\sigma }_{s}}={\frac{1-{\lambda }_{s}^{r}}{{P}_{a}}}\alpha_{s}{\theta }_{s}{\left(\frac{{\sigma }_{s}}{{P}_{a}}\right)}^{{\theta }_{s}-1}exp\left\{-{\alpha }_{s}{\left(\frac{{\sigma }_{s}}{{P}_{a}}\right)}^{{\theta }_{s}}-{\beta }_{s}{N}^{{\omega }_{s}}\right\} \;\; \mathrm{when } \;\; {d\sigma }_{s}>0$$

107 $$\frac{\partial {f}_{2}}{\partial {\sigma }_{s}}=0 \;\; \mathrm{when} \;\; d{\sigma }_{s}\le 0$$

108 $$\frac{\partial {f}_{2}}{\partial N}=\left(1-{\lambda }_{s}^{r}\right){\beta }_{s}{\omega }_{s}{N}^{{\omega }_{s}-1}exp\left\{-{\alpha }_{s}{\left(\frac{W}{{P}_{a}}\right)}^{{\theta }_{s}}-{\beta }_{s}{N}^{{\omega }_{s}}\right\}$$

##### Coefficient of local stress concentration

The local stress concentration coefficient establishes the relationship between the stress of cemented elements and root and the stress of representative elements. It is also an unmeasurable internal variable, which is related to the external load on the specimen and its internal stress–strain coordination ability. Although the bonded elements and the root have similar constitutive relationship, their local stress concentration coefficients are different because of their different elastic modulus and different strain coordination ability. Similar to the definition of breakage ratio, the local stress concentration coefficient can be divided into spherical stress concentration coefficient and deviatoric stress concentration coefficient. The stress concentration coefficient of bonded elements meets the requirement that when the breakage ratio is 0, the stress concentration coefficient is 1. Ignoring the influence of root, the simplified assumption is:

109 $${C}_{m}^{b}=1+{m}_{1}{\lambda }_{v}^{f}$$

110 $${C}_{s}^{b}=1+{m}_{2}{\lambda }_{s}^{f}$$

The volume of the root is constant during the stress loading process and the stress concentration coefficient of the root is simply assumed to be a variable related to the confining pressure.

### Model verification

Under triaxial test conditions, the mean stress, generalized shear stress, volumetric strain and shear strain have the following relations:

111 $${\varepsilon }_{v}={\varepsilon }_{1}+2{\varepsilon }_{3}$$

112 $${\varepsilon }_{s}=\frac{2}{3}\left({\varepsilon }_{1}-{\varepsilon }_{3}\right)$$

113 $${\sigma }_{m}=\frac{1}{3}\left({\sigma }_{1}+2{\sigma }_{3}\right)$$

114 $${\sigma }_{s}={\sigma }_{1}-{\sigma }_{3}$$

The actual number of freeze–thaw cycles is a discrete quantity, so it is verified under the same number of freeze–thaw cycles, that is, dN = 0. The above relation can be obtained by substituting it into Eqs. (54) and (55):

115 $${d\varepsilon }_{v}=d\left({\varepsilon }_{1}+2{\varepsilon }_{3}\right)={A}_{1}d\frac{1}{3}\left({\sigma }_{1}+2{\sigma }_{3}\right)+{B}_{1}d\left({\sigma }_{1}-{\sigma }_{3}\right)$$

116 $${d\varepsilon }_{s}=\frac{2}{3}d\left({\varepsilon }_{1}-{\varepsilon }_{3}\right)={A}_{2}d\frac{1}{3}\left({\sigma }_{1}+2{\sigma }_{3}\right)+{B}_{2}d\left({\sigma }_{1}-{\sigma }_{3}\right)$$

Due to ${d\sigma }_{3}=0$, we can get:

117 $$d{\varepsilon }_{3}=\frac{\left(\frac{{A}_{1}}{3}+{B}_{1}\right)-\frac{3}{2}\left(\frac{{A}_{2}}{3}+{B}_{2}\right)}{\left(\frac{{A}_{1}}{3}+{B}_{1}\right)+3\left(\frac{{A}_{2}}{3}+{B}_{2}\right)}{d\varepsilon }_{1}$$

118 $${d\sigma }_{1}=\frac{3}{\left(\frac{{A}_{1}}{3}+{B}_{1}\right)+3\left(\frac{{A}_{2}}{3}+{B}_{2}\right)}d{\varepsilon }_{1}$$

According to the triaxial test results, the basic parameters of the bonded elements, frictional elements and root were respectively determined by the above method, and then the internal state parameters were determined by the inversion of the triaxial test results. The parameters of the revised model of binary medium for soil samples containing roots under different confining pressures and different freeze–thaw cycles were determined as shown in Table 6.

**Table 6:** Parameter determination of extended binary medium model.

Parameter type	Parameter	Confining pressure	Parameter value
Bonded elements	${K}^{b}$ (kPa)		$187.4{P}_{a}{\left(\frac{{\sigma }_{3}}{{P}_{a}}\right)}^{0.5032}$
	${G}^{b}$ (kPa)		$112.4{P}_{a}{\left(\frac{{\sigma }_{3}}{{P}_{a}}\right)}^{0.5032}$
Frictional elements	${K}^{f}$ (kPa)		$73{P}_{a}{\left(\frac{{\sigma }_{3}}{{P}_{a}}\right)}^{0.3105}$
	${G}^{f}$ (kPa)		$21.3{\left(\frac{{\sigma }_{3}}{{P}_{a}}\right)}^{0.2223}$
	${\alpha }_{m}$		$0.9743{\left(\frac{{\sigma }_{3}}{{P}_{a}}\right)}^{-0.3184}$
	${c}_{1}$		$-0.3741\mathrm{ln}\left(0.1589\left(\frac{{\sigma }_{3}}{{P}_{a}}-0.2426\right)\right)-1.681$
	${c}_{2}$		$-0.07499{\left(\frac{{\sigma }_{3}}{{P}_{a}}\right)}^{-3.055}+0.3954$
	${p}_{0}$		200
	n		5
	n_1_		5
	$\psi$		0.0585
	κ		0.011
	${e}_{0}$		1.22
Root	${K}^{r}$(kPa)		135,000
	${G}^{r}$(kPa)		20,250
Breakage ratio parameter	${\alpha }_{v}$	25	$-0.01194\mathrm{ln}\left(0.8742N+0.000092\right)+0.139-0.005r$
		50	$0.1206\mathrm{ln}\left(0.7369N+1.95\right)+0.1632-0.015r$
		100	$0.0499\mathrm{ln}\left(0.1268\mathrm{N}+0.66\right)+0.2707-0.015r$
		200	$0.0539\mathrm{ln}\left(0.03369N+0.0542\right)+0.4075-0.015r$
	${\beta }_{v}$		$0.06841\mathrm{ln}\left(0.4938\frac{{\sigma }_{3}}{{P}_{a}}-0.0689\right)+0.2102$
	${\theta }_{v}$	25	$0.4321\mathrm{ln}\left(1.513N+0.7682\right)+2.119$
		50	$0.04871\mathrm{ln}\left(1.726N+1.334\right)+1.99$
		100	$0.09783\mathrm{ln}\left(1.136N+0.8878\right)+2.019$
		200	$0.5199\mathrm{ln}\left(1.523N+4.1578\right)+1.265$
	${\omega }_{v}$		0.25
	${\alpha }_{s}$		2
	${\beta }_{s}$		3
	${\theta }_{s}$		− 0.002
	${\omega }_{s}$		0.1
Stress concentration parameter	${m}_{1}$		− 0.4
	${m}_{2}$		− 0.3
	${C}_{m}^{r}$		$0.7981{\left(\frac{{\sigma }_{3}}{{P}_{a}}\right)}^{-0.1619}$
	${C}_{s}^{r}$		$0.8978{\left(\frac{{\sigma }_{3}}{{P}_{a}}\right)}^{-0.07796}$

**Figure 14:**
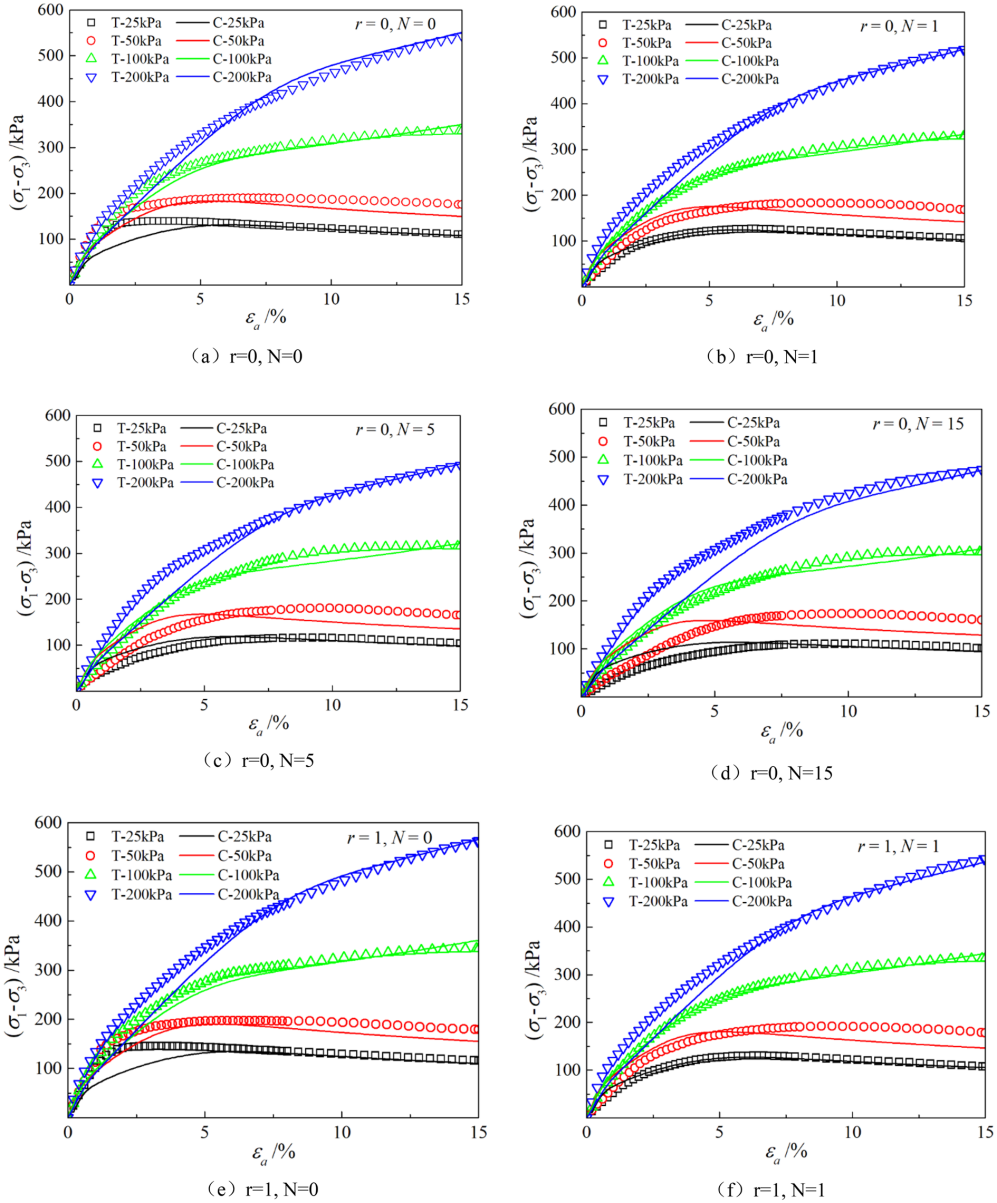

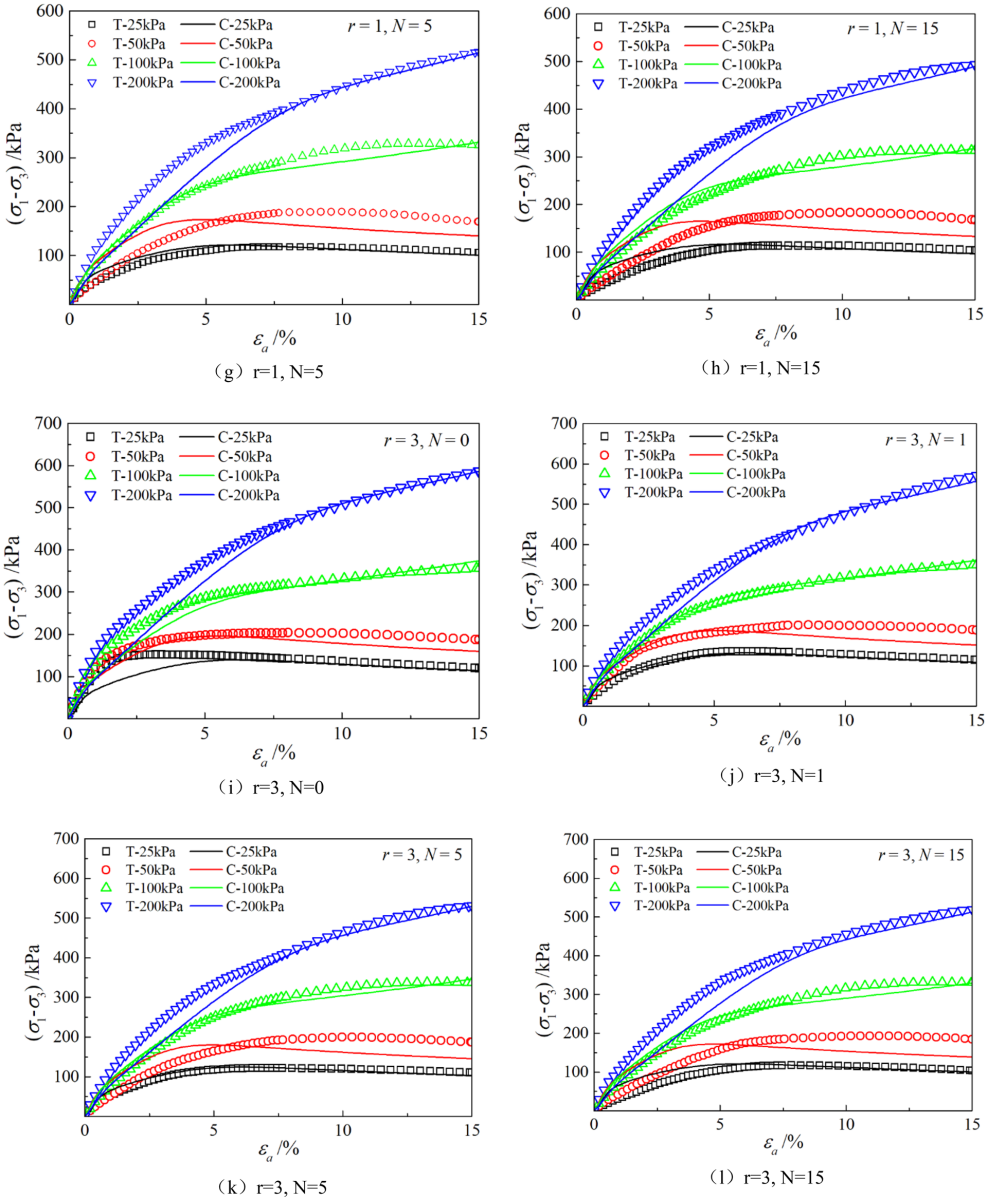
Comparison of ${\varepsilon }_{a}-\left({\sigma }_{1}-{\sigma }_{3}\right)$ curves under different confining pressures between test and simulation.

**Figure 15:**
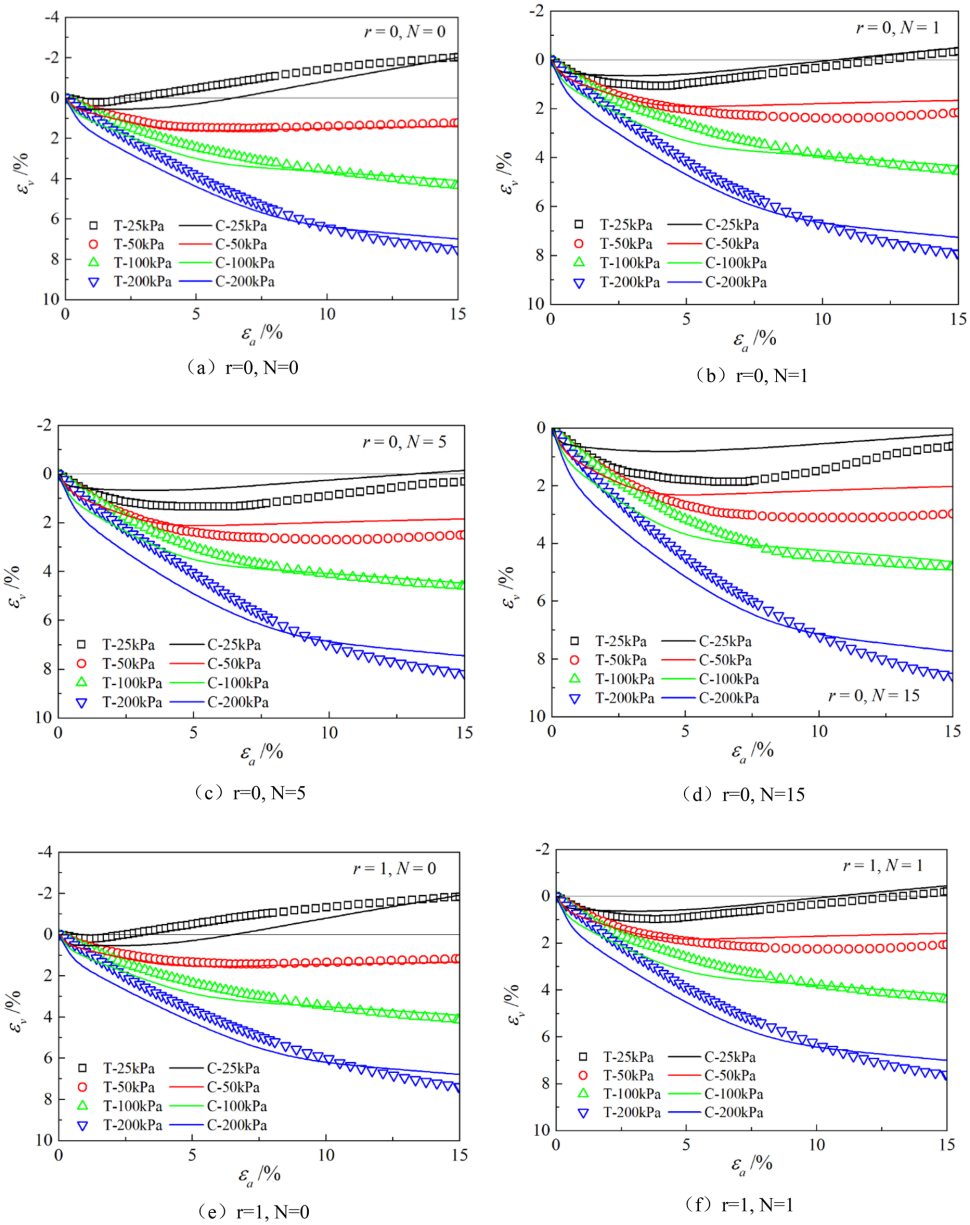

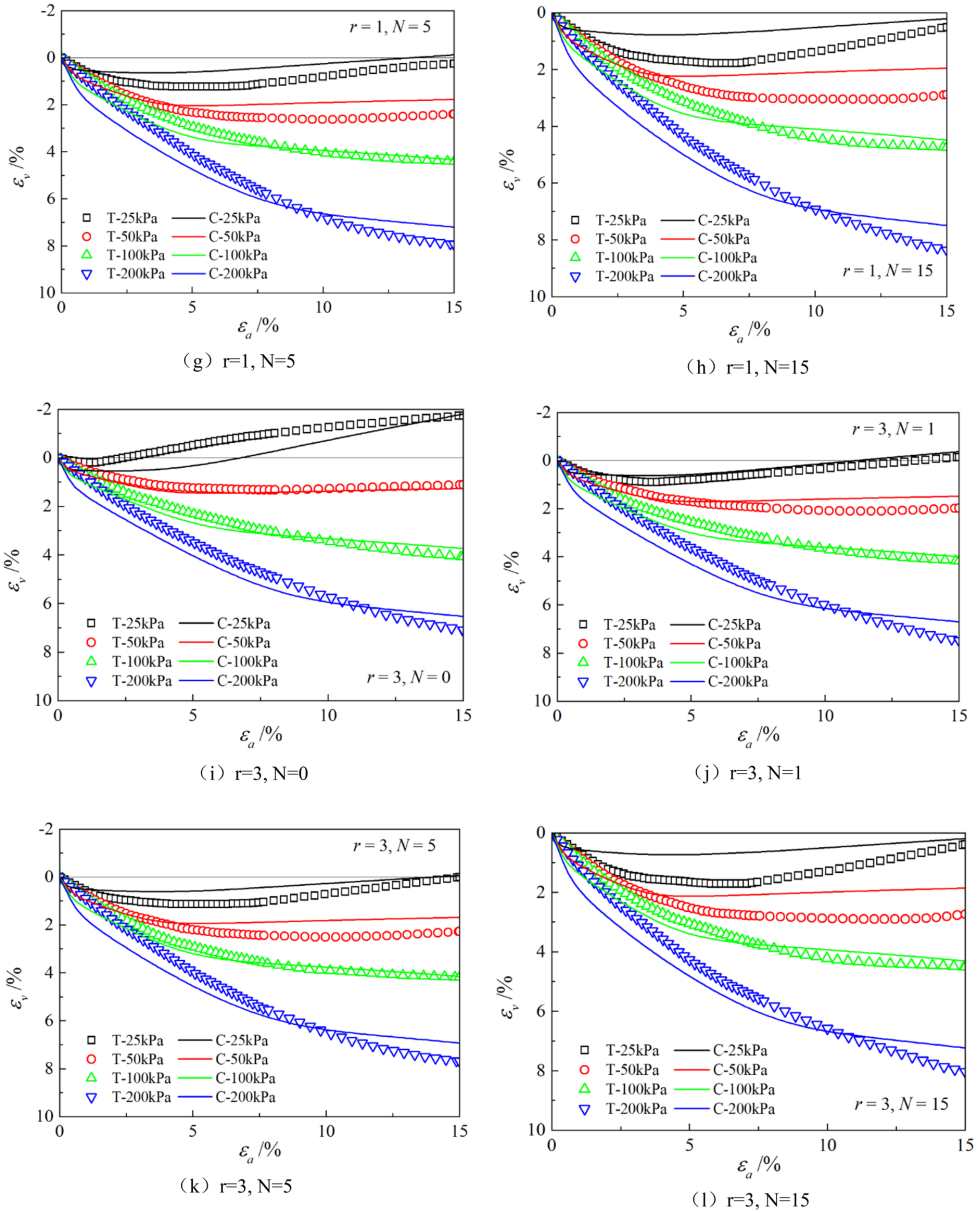
Comparison of ${\varepsilon }_{a}-{\varepsilon }_{v}$ curves under different confining pressures between test and simulation.

**Figure 16:**
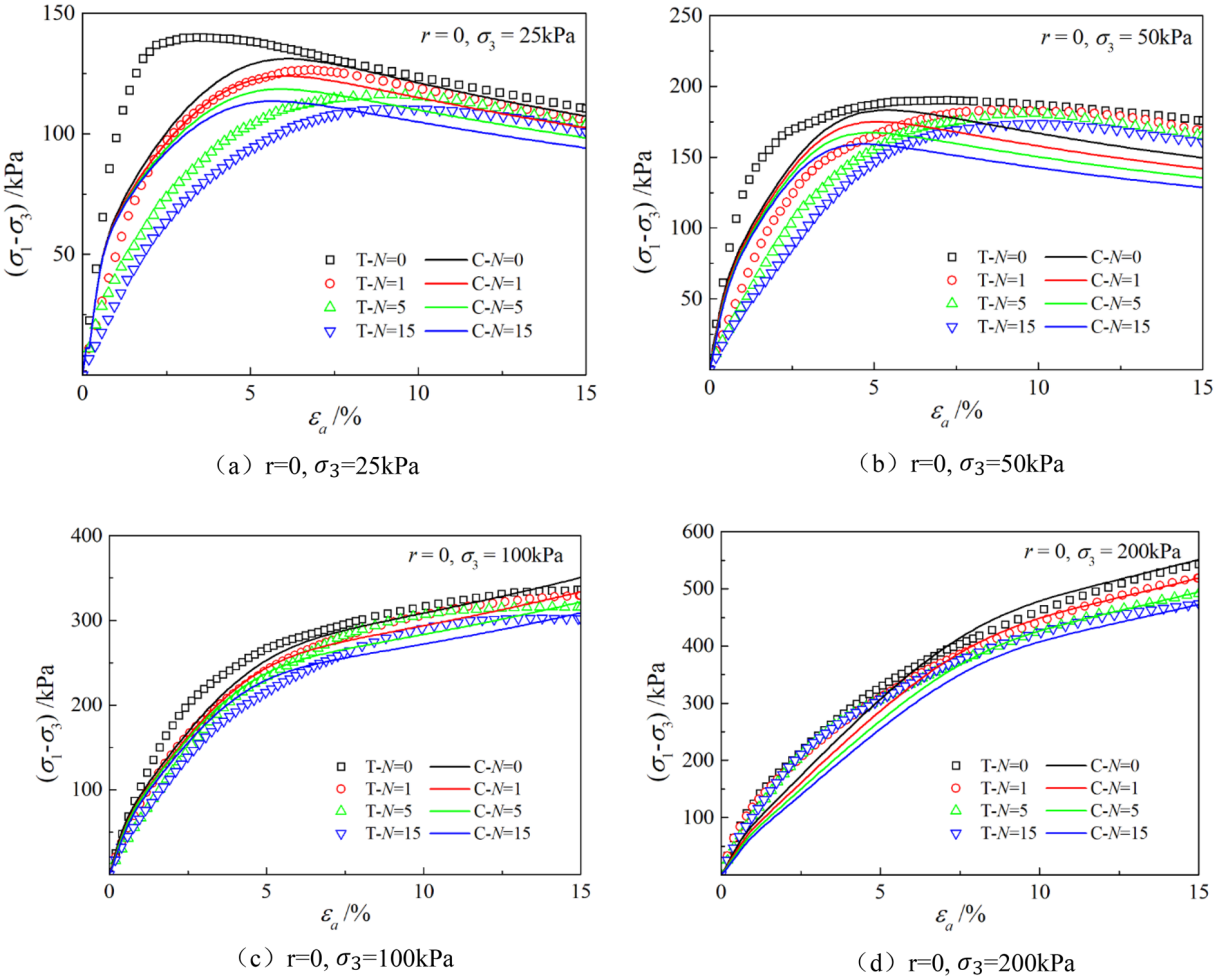
Comparison of ${\varepsilon }_{a}-\left({\sigma }_{1}-{\sigma }_{3}\right)$ curves under different freeze–thaw cycles between test and simulation.

**Figure 17:**
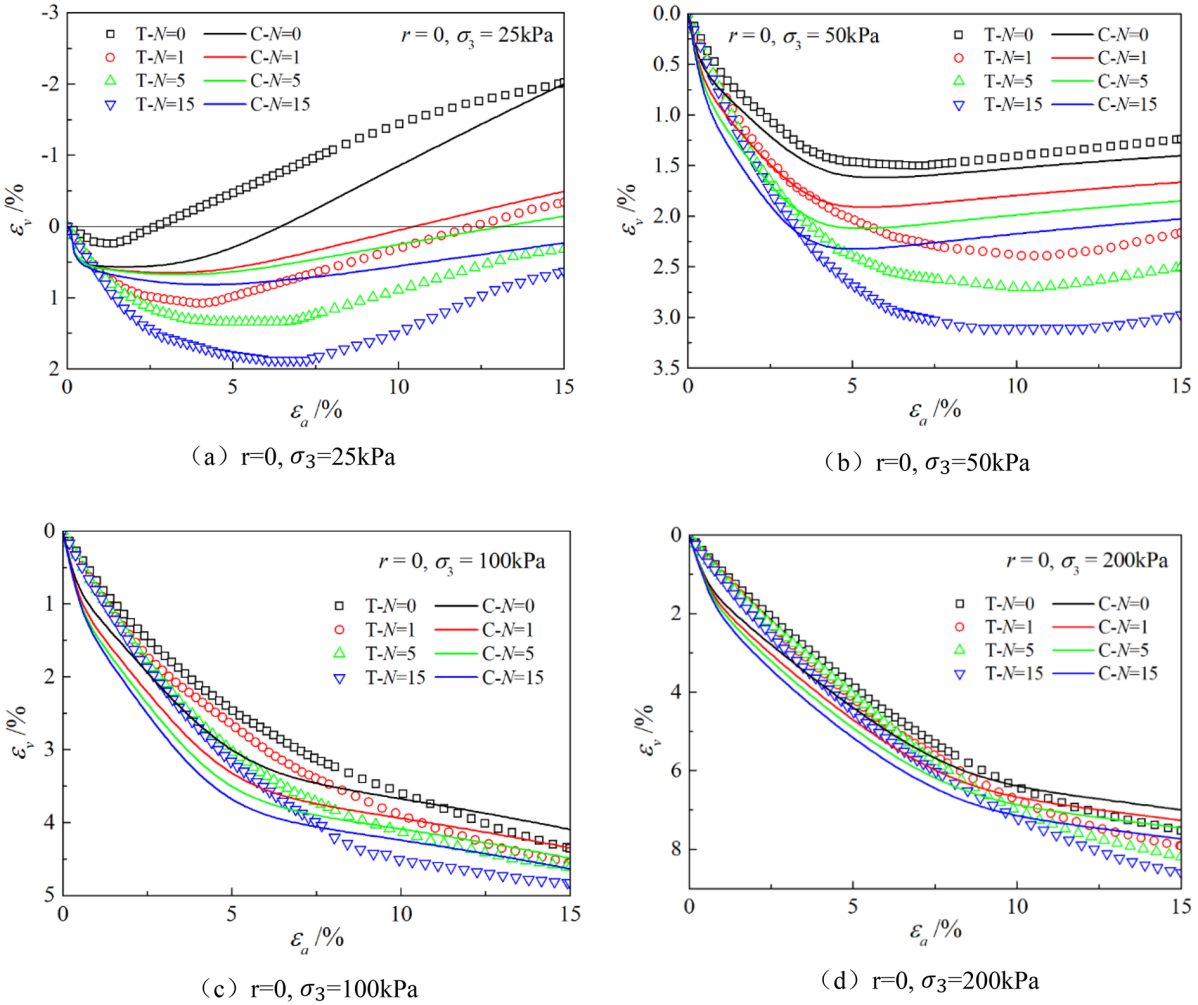
Comparison of ${\varepsilon }_{a}-{\varepsilon }_{v}$ curves under different freeze–thaw cycles between test and simulation.

**Figure 18:**
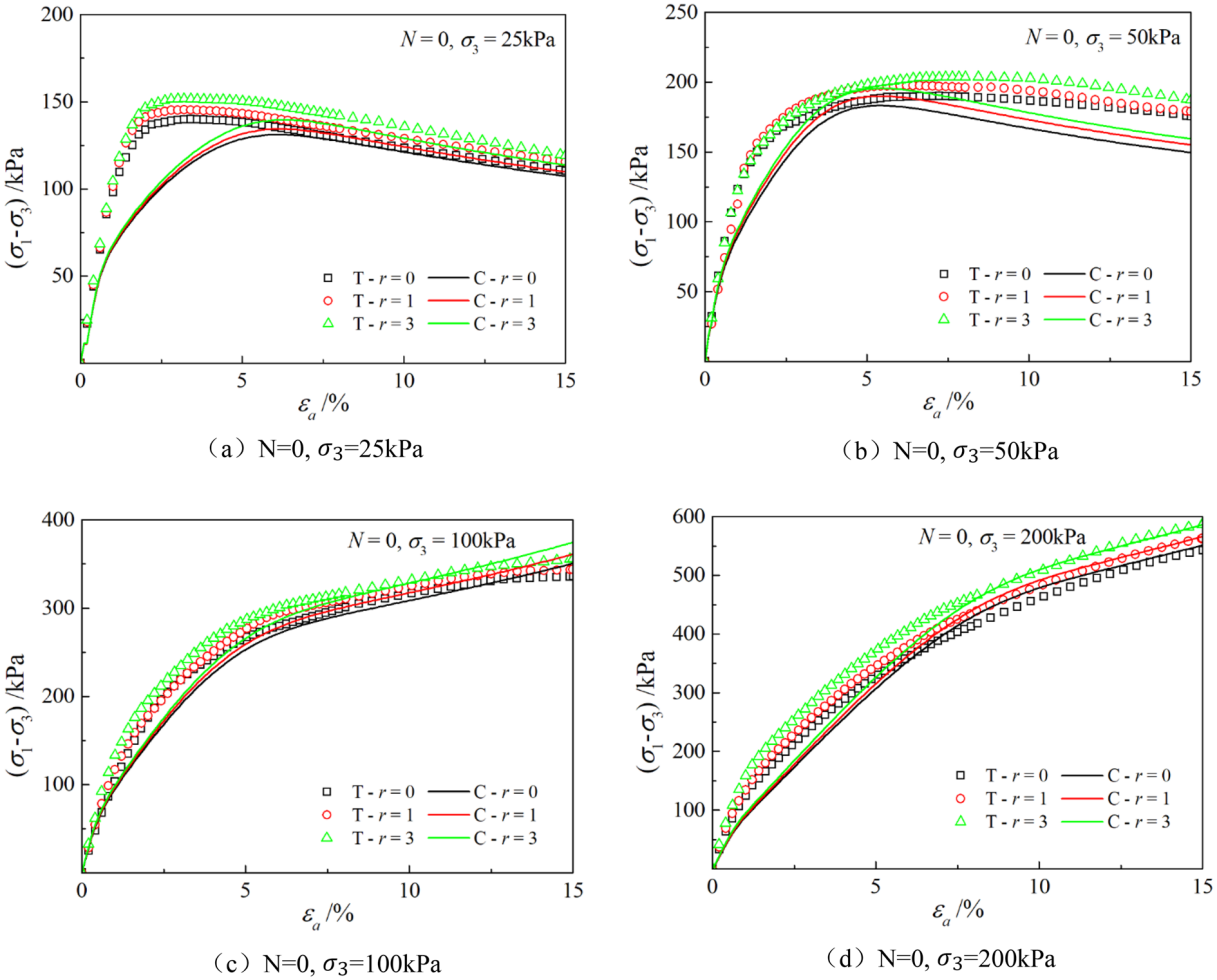
Comparison of ${\varepsilon }_{a}-\left({\sigma }_{1}-{\sigma }_{3}\right)$ curves with different roots between test and simulation.

**Figure 19:**
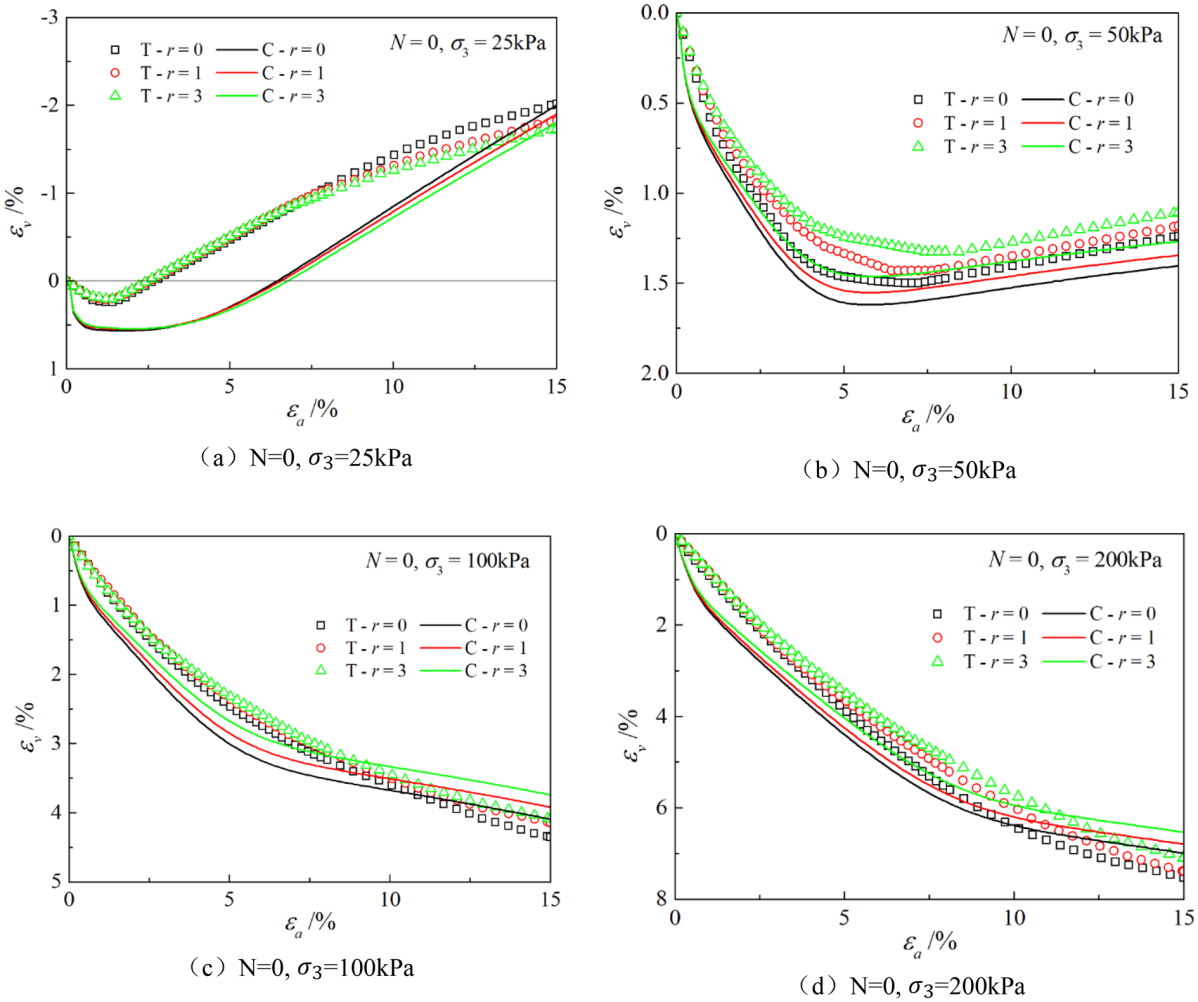
Comparison of ${\varepsilon }_{a}-{\varepsilon }_{v}$ curves with different roots between test and simulation.

The parameters determined in Table 6 were put into the stress–strain formula of the model for calculation, the test results were predicted, and curves ${\varepsilon }_{a}-\left({\sigma }_{1}-{\sigma }_{3}\right)$ and ${\varepsilon }_{a}-{\varepsilon }_{v}$ under different confining pressures and different times of freeze–thaw cycles with different volume ratio of roots were simulated. The simulation curves of part of the prediction are compared with the test curves, and the results are shown in Figs. 14, 15, 16, 17, 18, 19, where T represents the test and C represents the simulation calculation. It can be seen from the figure that the predicted results of the model are in good agreement with the triaxial test results of the samples, especially the strain softening and volumetric dilatancy phenomena of the samples can be simulated relatively well.

## Conclusions

In this paper, the stress–strain characteristics of rooted soil under the action of freeze–thaw cycle were experimentally studied, and the variation law of rooted soil with confining pressure, the number of freeze–thaw cycles and the percentage of rooted soil was obtained. And on the basis of the test, the rooted soil was abstracted into the bonded elements, frictional elements and root of the three-phase composite material, the extended binary medium model was derived and verified. The main conclusions of this paper are as follows:

1. Laboratory triaxial tests show that rooted soil exhibits strain softening and volumetric dilatancy at low confining pressures, and strain hardening and volumetric shrinkage at high confining pressures. During the freeze–thaw cycles, the cement structure in the soil is damaged by frost heave force. With the increase of freeze–thaw times, the bearing capacity of the sample gradually decreases, the deviatoric stress decreases, and the volumetric strain increases. The greater the root volume, the more the bearing capacity of the sample, and the smaller the root volume, so the strengthening effect is limited. Under the same other conditions, the sample strength increases with the increase of confining pressure, decreases with the increase of freeze–thaw times, and increases with the increase of root volume.
2. Based on the theory of breakage mechanics for geological materials, this paper abstracts soil into bonded elements that can be destroyed and frictional elements, and roots into bonded elements that will not be destroyed to jointly bear external load and deformation, and deduces a extended binary medium model of rooted soil under the action of freeze–thaw cycles. In this way, the binary medium model can be extended to the three-phase composite materials, which can adopt different constitutive relations. In this paper, linear elastic constitutive model (Hooke’s law) and double-hardening constitutive model are adopted respectively according to their respective characteristics. The simulation results and experimental comparison show that the model can simulate the mechanical properties of rooted soil under freeze–thaw cycle.
3. The extension from binary medium to three-phase material inevitably brings about an increase in the number of model parameters, so the parameters are not easy to determine and the error may increase, which requires more accurate and careful tests and as much as possible to establish more relationships to reduce parameters. In addition, the characteristics of concrete materials are more in line with the characteristics of this model. Aggregate is regarded as bonded elements that will not be destroyed, and cement mortar gradually breaks under external load and transforms from bonded elements to frictional elements. The applicability of this model to concrete can be further studied in the later stage.

## Data Availability

The datasets used and/or analyzed during the current study are available from the corresponding author on reasonable request.

## List of symbols

$${\sigma }_{ij}$$: Stress tensor
$${\varepsilon }_{ij}$$: Strain tensor
$${\delta }_{ij}$$: Kronecker function
V: Volume
*λ*: Breakage ratio
${\lambda }_{v}$: Volume breakage ratio
${\lambda }_{v}^{r}$: Volume ratio of roots to sample
${\lambda }_{s}$: Area breakage ratio
${\sigma }_{1}$,${\sigma }_{2}, {\sigma }_{3}$: Major, intermediate and minor principle stress, respectively
${\varepsilon }_{1}, {\varepsilon }_{2}, {\varepsilon }_{3}$: Major, intermediate and minor principle strain, respectively
$${\sigma }_{m}$$: Mean stress
${\sigma }_{s}$: Generalized shear stress
$${\varepsilon }_{v}$$: Volumetric strain
${\varepsilon }_{s}$: Generalized shear strain
$${\varepsilon }_{a}$$: Axial strain
*K*: Bulk modulus
*G*: Shear modulus
${K}_{v}^{f}, {K}_{s}^{f}, {G}_{v}^{f}, {G}_{s}^{f}$: Flexibility matrix components of the frictional elements
${C}_{m}$: Spherical stress concentration coefficient
${C}_{s}$: Deviatoric stress concentration coefficient
N: Number of freeze–thaw cycles
$$f$$: Yielding function
$$g$$: Plastic potential
$\alpha$,H: Hardening parameters of the frictional elements
${\alpha }_{m}$, c_1_, c_2_, *β*: State parameters of double hardening model
${p}_{0}$: Reference pressure on ${\varepsilon }_{v}^{fp}=0$
$\lambda^ {\prime}$: Virgin compression slope on ${\varepsilon }_{v}^{f}-\mathrm{ln}{p}^{f}$ plot
*κ*: Rebounding slope on ${\varepsilon }_{v}^{f}-\mathrm{ln}{p}^{f}$ plot
$$d\Lambda$$: Plastic multiplier
$$\nu$$: Poisson ratio
${e}_{0}$: Initial void ratio
${P}_{a}$: Standard atmospheric pressure
${m}_{1}{m}_{2}$: Stress concentration parameters
RVE: Representative volume element

## Superscripts

b: Bonded elements
f: Frictional elements
r: Roots
loc: The mesoscopic local
0: The current value of variables
e: Elastic
p: Plastic
no superscripts: The variable of RVE

## Code availability

Since this study did not use programming code, this section does not apply.

## References

[1] Guo Y (2013). The Mechanism of Cutting Slope Freeze–Thaw Instability and Its Protection by Vegetation in High-Latitude Frozen Regions.

[2] Yang YH, Wang CH, Liu SZ, Xiao QH (2007). Experimental research on improving shear strength of soil in surface landslide by root system of different vegetation type. Res. Soil Conserv..

[3] Skarzynska K. M. Formation of soil structure. In *Proceedings of the 4th International Symposium on Ground Freezing*, Vol. 2, 213–218 (1985)

[4] Konrad JM (1989). Physical processes during freeze-thaw cycles in clayey silts. Cold Reg. Sci. Technol..

[5] Eigenbrod KD (1996). Effects of cyclic freezing and thawing on volume changes and permeabilities of soft fine-grained soils. Can. Geotech. J..

[6] Simonsen E, Janoo VC, Isacsson U (2002). Resilient properties of unbound road materials during seasonal frost conditions. J. Cold Reg. Eng..

[7] Qi JL, Ma W, Song CX (2008). Influence of freeze-thaw on engineering properties of a silty soil. Cold Reg. Sci. Technol..

[8] Hanay A, Sahin U, AnaPali O (2010). Decrease in hydraulic conductivity of clay soils with salinity一sodicity problems due to freezing and thawing effect. Aeta Agric. Scand. Sect. B一Plant Soil Sci..

[9] Xu J, Li Y, Lan W (2019). Shear strength and damage mechanism of saline intact loess after freeze-thaw cycling. Cold Reg. Sci. Technol..

[10] Xu X, Zhang W, Wang Y (2022). Measuring and modeling the dielectric constant of soil during freezing and thawing processes: An application on silty clay. Acta Geotech.

[11] Wang B, Xu X, Wang X (2023). Mechanical behavior and strength criterion of frozen silty clay under complex stress paths. Geoderma.

[12] Benson CH, Abichou TH, Olson MA, Bosscher PJ (1995). Winter effects on hydraulic conductivity of compacted clay. J. Geotech. Eng. Eng..

[13] Bemand A, Mrrsion D, James M (1983). Changes in soil structure due to freeze-thaw and repeated loading. Transp. Res. Rec..

[14] Ono T. & Mitachi, T. Computer controlled triaxial freeze-thaw-shear apparatus. In *Proc. 8th Int. Symp. Ground Freezing*, 335–339 (A.A. Balkema, 1997)

[15] Graham J, Au VCS (1985). Effects of freeze–thaw and softening on a natural clay at low stresses. Can. Geotech. J..

[16] Wang DY, Ma W (2007). Effects of cyclic and thawing on mechanical properties of Qinhai-Tibet clay. Cold Reg. Sci. Technol..

[17] Broms BB, Yao LYC (1964). Shear strength of a soil after freezing and thawing. ASCE J. Soil Mech. Found. Div..

[18] Leroueil S, Tardif J, Roy M (2011). Effects of frost on mechanical behavior of Champlain Sea clays. Can. Geotech. J..

[19] Qin Z, Lai Y, Tian Y (2021). Effect of freeze-thaw cycles on soil engineering properties of reservoir bank slopes at the northern foot of Tianshan Mountain. J. Mt. Sci..

[20] Yong R. N., Boonsinsuk P. & Yin, C. W. P. Alternation of soil behavior after cyclic freezing and thawing. In *Proceedings of 4th international Symposium on Ground Freezing*, 187–195 (A.A. Balkema, 1985)

[21] Bondarenko, G. I. & Sadovsky, A. V. Water content effect of the thawing clay soils on shear strength. In* Proceedings of 7th International Symposium on Ground Freezing*, 123–127 (A. A. Balkema, 1991)

[22] Christopher S, Christopher G (1998). Freeze-thaw effects on Boston Blue clay. J. Eng. Appl. Sci. Soil Improv. Big Digs.

[23] Fang H, Lin JP (2004). Slope vegetation: Present situation and its prospect. Res. Soil Conserv..

[24] Neto MIM, Mahler CF (2017). Study of the shear strength of a tropical soil with grass roots. Soils Rocks.

[25] Valizade N, Tabarsa A (2022). Laboratory investigation of plant root reinforcement on the mechanical behaviour and collapse potential of loess soil. Eur. J. Environ. Civ. Eng..

[26] Genet M, Stokes A, Salin F (2005). The influence of cellulose content on tensile strength in tree roots. Plant Soil.

[27] Tosi M (2007). Root tensile strength relationships and their slope stability implications of three shrub species in Northern Apennines (Italy). Geomorphology.

[28] Gray DH, Sotir RB (1996). Biotechnical and Soil Bioengineering Slope Stabilization: A Practical Guide for Erosion Control.

[29] Wu T. H. *Investigation of Landslides on Prince of Wales Island, Alaska*. Rpt. Vol. 5, 93 (Ohio State Univ., Dept. of Civil Eng., Geotech. Eng., 1976)

[30] Wu TH, McKinnell WP, Swanston DN (1979). Strength of tree roots and landslides on Prince of Wales Island, Alaska. Can. Geotech. J..

[31] Pollen N, Simon A (2005). Estimating the mechanical effects of riparian vegetation on stream bank stability using a fiber bundle model. Water Resour..

[32] Schwarz M, Lehmann P, Or D (2010). Quantifying lateral root reinforcement in steep slopes—From a bundle of roots to tree stands. Earth Surf. Process Landforms.

[33] Lee EH, John BJ (2009). Examining the impact of wind and surface vegetation on the Asian dust occurrence over three classified source regions. J. Geophys. Res. Atmos..

[34] Chirico GB, Borgab M, Tarollib P, Rigonc R, Pretid F (2013). Role of vegetation on slope stability under transient unsaturated conditions. Procedia Environ. Sci..

[35] Ng CWW (2017). Atmosphere-plant-soil interactions: Theories and mechanisms. Chin. J. Geotech. Eng..

[36] Shen ZJ (2002). Breakage mechanics and double-medium model for geological materials. Hydrosci. Eng..

[37] Shen ZJ, Liu EL, Chen TL (2005). Generalized stress-strain relationship of binary medium model for geological materials. Chin. J. Geotech. Eng..

[38] Shen, Z. J. Progress in binary medium modeling of geological materials. In *Modern trends in Geomechanics, Springer Proceedings in Physics*, (eds Wu, W. & Yu, H.-S.) 77–99 (Springer, 2006)

[39] Liu EL (2006). Study on Breakage Mechanism of Structural Blocks and the Binary-Medium Model for Geomaterials.

[40] Liu EL, Huang RQ, He SM (2012). Modeling the deformation properties of rock samples by Binary Medium Model. J. Hydraul. Eng..

[41] Zhang D, Liu EL (2019). Binary-medium-based constitutive model of frozen soils subjected to triaxial loading. Results Phys..

[42] Liu Y, Liu E, Yin Z (2020). Constitutive model for tailing soils subjected to freeze–thaw cycles based on meso-mechanics and homogenization theory. Acta Geotech..

[43] Yu D, Liu E, Xiang B (2023). A micro–macro constitutive model for rock considering breakage effects. Int. J. Min. Sci. Technol..

[44] Chen Y, Liu E, Yu Y (2023). A binary-medium-based constitutive model for porous rocks. Int. J. Rock Mech. Min. Sci..

[45] Wang, P. *et al.* A rate-dependent constitutive model for saturated frozen soil considering local breakage mechanism. *J. Rock Mech. Geotech. Eng.* (2023).

[46] Tian J, Lai Y, Liu E, He C (2023). A thermodynamics-based micro-macro elastoplastic micropolar continuum model for granular materials. Comput. Geotech..

[47] Shen Z (1995). A double hardening model for clays. Rock Soil Mech..

[48] Liu EL, Xing HL (2009). A double hardening thermo-mechanical constitutive model for overconsolidated clays. Acta Geotech..

